# Cytosolic *O*-GlcNAcylation and PNG1 maintain *Drosophila* gut homeostasis by regulating proliferation and apoptosis

**DOI:** 10.1371/journal.pgen.1010128

**Published:** 2022-03-16

**Authors:** Hyun-jin Na, Lara K. Abramowitz, John A. Hanover

**Affiliations:** Laboratory of Cellular and Molecular Biology, National Institute of Diabetes and Digestive and Kidney Diseases, National Institutes of Health, Bethesda, Maryland, United States of America; Capital Normal University, CHINA

## Abstract

Tissue homeostasis requires a delicate balance between stem cell self-renewal, proliferation, and differentiation. Essential to this process is glycosylation, with both intra-and extra-cellular glycosylation being required for stem cell homeostasis. However, it remains unknown how intracellular glycosylation, *O*-GlcNAcylation, interfaces with cellular components of the extracellular glycosylation machinery, like the cytosolic *N-*glycanase NGLY1. In this study, we utilize the *Drosophila* gut and uncover a pathway in which *O*-GlcNAcylation cooperates with the NGLY1 homologue PNG1 to regulate proliferation in intestinal stem cells (ISCs) and apoptosis in differentiated enterocytes. Further, the CncC antioxidant signaling pathway and ENGase, an enzyme involved in the processing of free oligosaccharides in the cytosol, interact with *O*-GlcNAc and PNG1 through regulation of protein aggregates to contribute to gut maintenance. These findings reveal a complex coordinated regulation between *O*-GlcNAcylation and the cytosolic glycanase PNG1 critical to balancing proliferation and apoptosis to maintain gut homeostasis.

## Author summary

Understanding glycosylation is of great importance as changes in both intra- and extra-cellular glycosylation have been well documented in diseases. However, it remains relatively unclear how intracellular glycosylation, *O*-GlcNAcylation, interfaces with the cellular machinery of extracellular glycosylation. In this study, we seek to understand how coordination between a cytosolic N-glycanase, NGLY1, and intracellular glycosylation, is utilized during stem cell proliferation. Using the *Drosophila* gut as a model system, our data showed *O*-GlcNAcylation and the *Drosophila* homologue of NGY1, PNG1, have key roles in both progenitor and differentiated cells that contribute to tissue homeostasis. Further, the CncC antioxidant signaling pathway and ENGase, an enzyme involved in the processing of free oligosaccharides in the cytosol, interact with *O*-GlcNAc and PNG1 through regulation of protein aggregates to contribute to gut maintenance. Our study suggests that alterations in *O*-GlcNAc and NGLY1 could be future targets utilized as therapeutics for nutrient-sensitive stem cell-derived diseases such as cancer.

## Introduction

Glucose can be metabolized through multiple cell signaling pathways, including glycolysis, glycogen synthesis, and the hexosamine biosynthetic pathway (HBP) [1]. The HBP plays a critical role in nutrient sensing, stress response, cell growth, and organ development [1]. Nutrient availability controls HBP flux ultimately impacting protein *O-*GlcNAcylation as well as *N-* and *O-*glycosylation [2,3].

Glycosylation plays essential roles in cell pluripotency, embryogenesis, cell-to-cell interaction, and cell-to-environment interactions, ultimately influencing cellular processes such as protein folding and signal transduction [4–7]. Understanding regulation of glycosylation and the interaction between intra- and extra-cellular glycosylation is of great importance as changes in glycosylation have been well documented in various diseases. Deregulation of intracellular *O-*linked glycosylation, *O-*GlcNAc, as well as the enzymes involved in adding this modification to proteins (*O-*GlcNAc Transferase, OGT) and removing this glycosylation (*O-*GlcNAcase, OGA) have been described in numerous types of cancers [8]. Cancer stem cells often have alterations in glycan length toward shorter *O-*glycans and more branched *N-*glycans [9,10]. Further, aberrant glycosylation is closely linked to tumor progression by regulating tumor proliferation. In fact, alterations in glycosylation have been called a hallmark of cancer [10,11]. Thus, understanding the enzymes that remove glycans, glycanases, can prove essential to disease treatment [10]. Our previous work uncovered the role of *O*-GlcNAcase in the stress response to maintain gut homeostasis in *Drosophila*. Building on that work, here we assess the coordination between a cytosolic *N-*glycanase, NGLY1, and intracellular glycosylation in maintaining gut homeostasis through balancing proliferation and apoptosis.

NGLY1 encodes an evolutionarily conserved enzyme that catalyzes the cleavage of *N-*glycans from glycoproteins in the cytosol [12], playing a crucial role for newly synthesized *N-*glycoproteins [13]. NGLY1 defects are likely to affect the quality control and homeostasis of many cellular proteins, subsequently perturbing signaling pathways, cell physiology, and organ development [12]. Patients who are NGLY1-deficient display global developmental delay, movement disorder and growth retardation [14,15]. Loss-of-function mutants of *Drosophila* PNGase (PNG1; peptide: *N-*glycanase; NGLY1 in human/mice) caused semi-lethality and sterility [16]. NGLY1 removes *N-*glycans from misfolded glycoproteins during endoplasmic reticulum (ER)-associated degradation and is thought to play an important role in the efficient degradation of misfolded glycoproteins [17–19]. Not only is NGLY1 essential in a variety of cells, it plays a crucial role in cancer development by being expressed in many types of cancer cells [20,21]. Despite its essential function in normal development and disease progression, the biological functions of NGLY1 in highly proliferating stem cells remains unclear.

**Fig 1:**
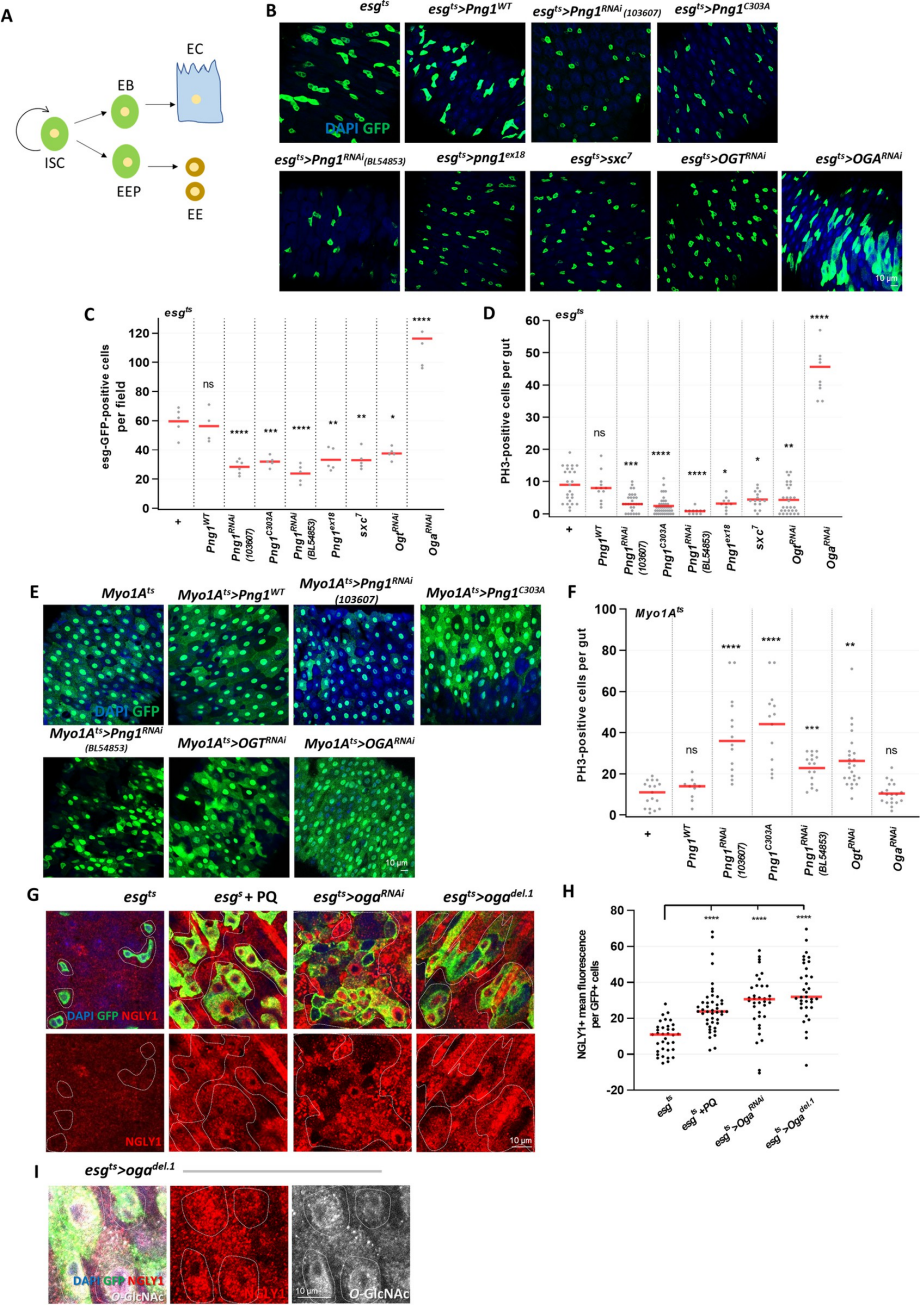
PNG1 regulates stem cell proliferation and is elevated in ROS-induced and OGA knockdown midgut ISCs/EBs. (A) Model of the ISC regeneration and lineage specification process. (B) The midgut *of esg*^*ts*^*>+*, *esg*^*ts*^*>Png1*^*wt*^, *esg*^*ts*^*>Png1*^*RNAi*^ (103607), *esg*^*ts*^*>Png1*^*C303A*^, *esg*^*ts*^*>Png1*^*RNAi*^ (BL54853), *esg*^*ts*^*>Png1*^*EX18*^, *esg*^*ts*^*>sxc*^*7*^, *esg*^*ts*^*>Ogt*^*RNAi*^, and *esg*^*ts*^*>Oga*^*RNAi*^ flies. (C) The number of esg-GFP-positive cells per field. (D) The number of PH3-positive cells in midguts from flies of the indicated genotype. (E) Imaging of Myo1A-GFP (green) in midgut of *Myo1A*^*ts*^*>+*, *Myo1A*^*ts*^*>Png1*^*wt*^, *Myo1A*^*ts*^*>Png1*^*RNAi*^ (103607), *Myo1A*^*ts*^*>Png1*^*C303A*^, *Myo1A*^*ts*^*>Png1*^*RNAi*^ (BL54853), *Myo1A*^*ts*^*>Ogt*^*RNAi*^, and *Myo1A*^*ts*^*>Oga*^*RNAi*^ flies. (F) The number of PH3-positive cells in midguts from flies of the indicated genotype. (G) Immunofluorescence staining of NGLY1 (red) in esg-GFP-positive cells (green) in midgut of 7-day-old *esg*^*ts*^*>+*, PQ-treated *esg*^*ts*^*>+*, *esg*^*ts*^*>Oga*^*RNAi*^, and *esg*^*ts*^*;Oga*^*del*.*1*^ flies. (H) Quantification of NGLY1 mean fluorescence per esg-GFP-positive cell. (I) Immunofluorescence staining of NGLY1 (red) and *O-*GlcNAc (white) in esg-GFP-positive cells (green) from the midgut of *esg*^*ts*^*;Oga*^*del*.*1*^ flies. Outline indicates esg-positive cells. Data are represented as mean ± SD. *p< 0.05. **p< 0.01. ***p< 0.001. ****p< 0.0001. n.s., not significant., see S1 Table for N values.

Adult stem cells play essential roles in tissue function and development by providing a reservoir of cells for homeostasis and regeneration [22]. The *Drosophila* intestine has proven to be an excellent model system to study how adult stem cell proliferation and differentiation are regulated and share many similarities in terms of development, cellular make-up and genetic control with mammals [23]. The adult midgut epithelium is composed of 4 different cell types: intestinal stem cells (ISCs), undifferentiated progenitor cells called enteroblasts (EBs) and specialized absorptive enterocytes (ECs) and secretory enteroendocrine cells (EEs) [24,25] (Fig 1A). The *Drosophila* intestine has proven to be an excellent model system to study how adult stem cell proliferation and differentiation are regulated and share many similarities in terms of development, cellular make-up and genetic control with mammals. Both ISCs and EBs express the SNAIL family transcription factor escargot (esg). Polyploid ECs, are characterized by the expression of Myosin31DF (Myo1A), differentiate from EBs. EEs, marked by the expression of prospero (pros), are derived from ISCs through distinct progenitors, called pre-EEs, that express Piezo, a cation channel that senses mechanical tension [24,25]. Previous studies have identified the HBP as a key player regulating ISC response to nutrition and midgut adaptation [26,27]. We have previously investigated the role of *O-*GlcNAcylation in ISCs/EBs through genetic mutation of the enzymes involved in *O-*GlcNAc cycling, OGT and OGA. We reported that *O-*GlcNAcylation controls *Drosophila* intestinal stem cell/progenitor cell homeostasis [27]. These data indicated that *O-*GlcNAc is important for stem cell maintenance, function, and tissue development through regulation of the DNA damage response and stress-induced proliferation [27]. However, it remains unknown if intracellular glycosylation interfaces with cytosolic glycanases like NGLY1 in adult stem cells, and further how this interaction is utilized in response to stress to maintain normal gut homeostasis.

In this study we uncover a pathway by which loss of PNG1 interacts with the enzymes of *O*-GlcNAc cycling in ISC/EBs and further differentiated EC cells to maintain gut homeostasis. Genetic analysis suggests that *O*-GlcNAc and NGLY1 act within the same pathway to regulate proliferation in ISC/EBs and apoptosis in ECs. Surprisingly, we find coordinate regulation of PNG1 and OGT, with PNG1 acting to stabilize OGT. Further investigation revealed that modulation of Nrf2 antioxidant signaling acts downstream of PNG1 and OGT to regulate the proliferation/apoptosis balance required in ISC/EBs and ECs for tissue maintenance. Similarly, Endo-beta-*N-*acetylglucosaminidase (ENGase) which is a key enzyme involved in the processing of free oligosaccharides in the cytosol, also participates in maintaining gut homeostasis through the PNG1/OGT pathway. Thus, abnormal phenotypes of OGT/PNG1 knockdown can be rescued by their downstream effectors, CncC and ENGase through regulation of protein aggregates and proteasome activity. The results highlight the importance of glycosylation in nutrient-sensitive stem cell diseases like cancer. We also present an underlying molecular mechanism and unexpected pathway that can be targeted for treating NGLY1-deficient patients.

## Results

### PNG1 regulates stem cell proliferation and is elevated in ROS-induced and OGA knockdown midgut ISCs/EBs

Previous reports indicate that Both PNG1 (NGLY1) and OGT have critical roles within the gut that contribute to normal gut homeostasis and importantly the overall health of the organism. PNG1 null larvae have specific developmental abnormalities in their midgut that contributes to their lethality [28]. Further, intestinal inflammation in Crohn’s disease is associated with increased *O*-GlcNAc modification [29]. Our previous study also showed that increased *O*-GlcNAc promotes gut dysplasia through regulation of DNA damage [27]. Thus, Png1 or *O*-GlcNAc might be associated with gut dysfunction in a disease context. To define the interaction between intracellular glycosylation and the cytosolic glycosidase NGLY1 we first investigated if the *Drosophila* homologue of NGLY1, PNG1, contributes to proliferation in adult stem cells. We used flies in which the enzyme PNG1 was knocked down (*UAS-Png1*^*RNAi*^ #103607; *UAS-Png1*^*RNAi*^ #BL54853*)*, mutated (UAS*-Png1*^*C303A*^ and *Png1*^*ex18*^), or Wild-Type (*UAS*-*Png1*^*WT*^) specifically in ISCs/EBs (*esg*^*ts*^, ISC/EB cell-specific inducible GAL4) or ECs (*Myo1A*^*ts*^,UAS-GFP,Gal4; EC-cell-specific inducible GAL4). After incubating at 29°C, we observed that GFP-positive cells (indicating ISC/EB) and PH3-positive cells (phospho-histone H3; a marker of cell proliferation) of *esg*^*ts*^*>Png1*^*WT*^ did not significantly change compared to control (Fig 1B–1D). However, there was a significant decrease in the number of GFP-positive and PH3-positive cells in the *esg*^*ts*^*>Png1*^*RNAi*^, *esg*^*ts*^*>Png1*^*C303A*^ and *esg*^*ts*^*>Png1*^*ex18*^ midguts compared to control (Fig 1B–1D). Interestingly, in the EC-specific PNG1 knockdown (*Myo1A*^*ts*^*>Png1*^*RNAi*^) and PNG1 mutant midguts (*Myo1A*^*ts*^*>Png1*^*C303A*^), the number of PH3-positive cells were significantly increased (Fig 1E and 1F). As shown in S1 Fig, ISC proliferation was induced while mature EC cells were reduced in EC-specific Png1 knockdown midgut compared to control. The percentage of Delta positive cells (ISCs) vs. Myo1A-GFP positive cells (mature cells) increased in Png1 knockdown midgut compared to control. These phenotypes are similar to the ISC/EB- and EC-specific OGT knockdown phenotypes described previously [27], and confirmed here (Fig 1B–1F). Thus, our data indicated that loss of PNG1 in ISCs/EBs suppressed ISC proliferation and loss of PNG1 in ECs promoted ISC hyperproliferation, similar to what was observed with loss of OGT. Because the PNG1 and OGT mutant phenotypes looked similar, we next investigated the association between PNG1 levels and *O-*GlcNAcylation. We assessed PNG1 levels in Paraquat (PQ)-treated (an inducer of extrinsic oxidative stress), OGA knockdown, and OGA del.1 mutant midgut ISCs/EBs, all of which have been shown to elevate *O-*GlcNAc levels [27]. We found that PNG1 levels were significantly increased, concomitant with *O-*GlcNAc in the PQ-treated, OGA knockdown, and OGA del.1 mutant midgut ISCs/EBs (Fig 1G–1I). We next assessed ISC proliferation in PNG1 mutants (*Png1*^*ex18*^; FRT82B) and OGT mutants (*sxc*^*7*^; FRT82B) using the MARCM (mosaic analysis with a repressible cell marker) system. We found that the number of clones and clone size decreased in *Png1*^*ex18*^ and *sxc*^*7*^ mutant midguts (S2A–S2C Fig). *O*-GlcNAc and PNG1 were reduced in *Png1*^*ex18*^ and *sxc*^*7*^ mutant midguts (S2D and S2E Fig). Additionally, our previous data showed that ISC proliferation and *O*-GlcNAc levels increased in OGA^del.1^, FRTB2B mutant ISCs compared to control [27]. Therefore, our data revealed that increased *O-*GlcNAcylation is correlated with an increase in PNG1 levels.

### PNG1 knockdown rescues dysplasia induced by hyper-*O*-GlcNAcylation in ISCs/EBs

**Fig 2:**
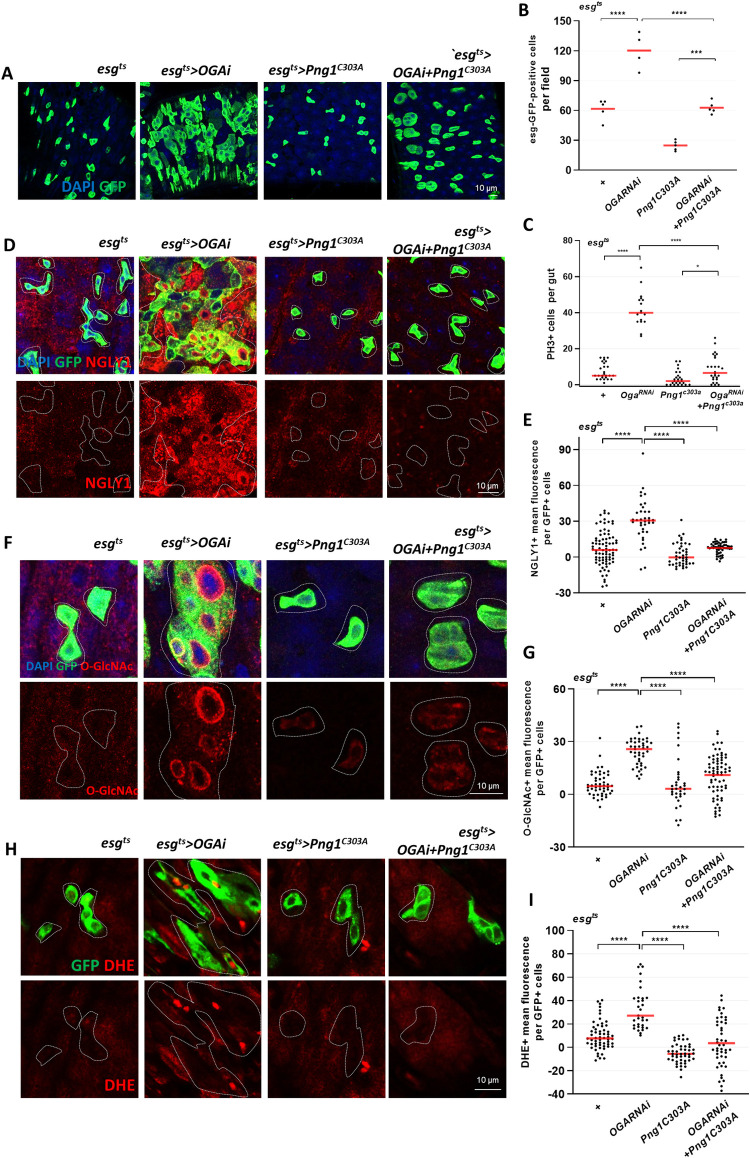
PNG1 knockdown rescues dysplasia induced by hyper-*O-*GlcNAcylation in ISCs/EBs. (A) After 7 days incubation at 29°C, the midgut of *esg*^*ts*^*>Oga*^*RNAi*^, *esg*^*ts*^*>Png1*^*C303A*^, and *esg*^*ts*^*>Oga*^*RNAi*^*+Png1*^*C303A*^ flies. (B) The number of esg-GFP-positive cells per field. (C) The number of PH3-positive cells in midguts from flies of the indicated genotype. (D) Immunofluorescence staining of NGLY1 (red) in esg-GFP-positive cells (green) in midgut of flies from the indicated genotype. (E) Quantification of NGLY1 mean fluorescence from the indicated genotype. (F) Immunofluorescence staining of *O-*GlcNAc (red) in esg-GFP-positive cells (green) in midgut of flies from the indicated genotype. (G) Quantification of *O-*GlcNAc mean fluorescence from the indicated genotype. (H) DHE staining (red) in midgut of flies. (I) Quantification of DHE mean fluorescence per esg-GFP-positive cell from the indicated genotype. Outline indicates esg-positive cells. Data are represented as mean ± SD. *p< 0.05. ****p< 0.0001., see S1 Table for N values.

Our data suggested that PNG1 levels increased in OGA knockdown and OGA del.1 mutant midgut ISCs/EBs. Additionally, the increased PNG1 levels significantly correlated with *O-*GlcNAc in ISCs/EBs (Fig 1), suggesting a link between intracellular *O*-GlcNAc and PNG1. Thus, we hypothesized an interaction between *O-*GlcNAc and PNG1 in stem cells and progenitor cells. To test this hypothesis, we asked if the mutant *Png1*^*C303A*^ could rescue ISC hyperproliferation, increased *O-*GlcNAcylation and elevated ROS levels in our ISC/EB-specific OGA knockdown. Our data confirmed ISC hyperproliferation in OGA knockdown ISCs/EBs [27] (Fig 2A–2C). Next, we assessed ISC proliferation rate through quantification of GFP-positive and PH3-positive cells and found that the double knockdowns rescued the hyperproliferation observed in the OGA single-knockdown midguts (Fig 2A–2C). These findings suggested that PNG1 was required for ISC hyperproliferation in the OGA knockdown midguts. Next, we assessed *O-*GlcNAc and PNG1 levels in the double-mutant fly midguts. There were high levels of *O-*GlcNAc and PNG1 staining in the single OGA knockdown and low levels of staining in the *Png1*^*C303A*^ single mutant midgut ISCs/EBs compared to control (Fig 2D–2G). We found a significant decrease in both *O-*GlcNAc and PNG1 levels in *esg*^*ts*^*>OGA*^*RNAi*^*+Png1*^*C303A*^ midgut ISCs/EBs compared to *esg*^*ts*^*>OGA*^*RNAi*^ ISCs/EBs (Fig 2D–2G). Additionally, the elevated DHE (dihydroethidium) signal (ROS detection marker) detected in the single OGA-RNAi mutant was rescued when combined with the *Png1*^*C303A*^ mutant in ISCs/EBs (Fig 2H and 2I). Therefore, OGA knockdown-induced ISC proliferation, increased PNG1, and elevated ROS levels were rescued by loss of PNG1 function in ISCs/EBs.

### Regulation of PNG1 and OGT levels in ISCs/EBs

**Fig 3:**
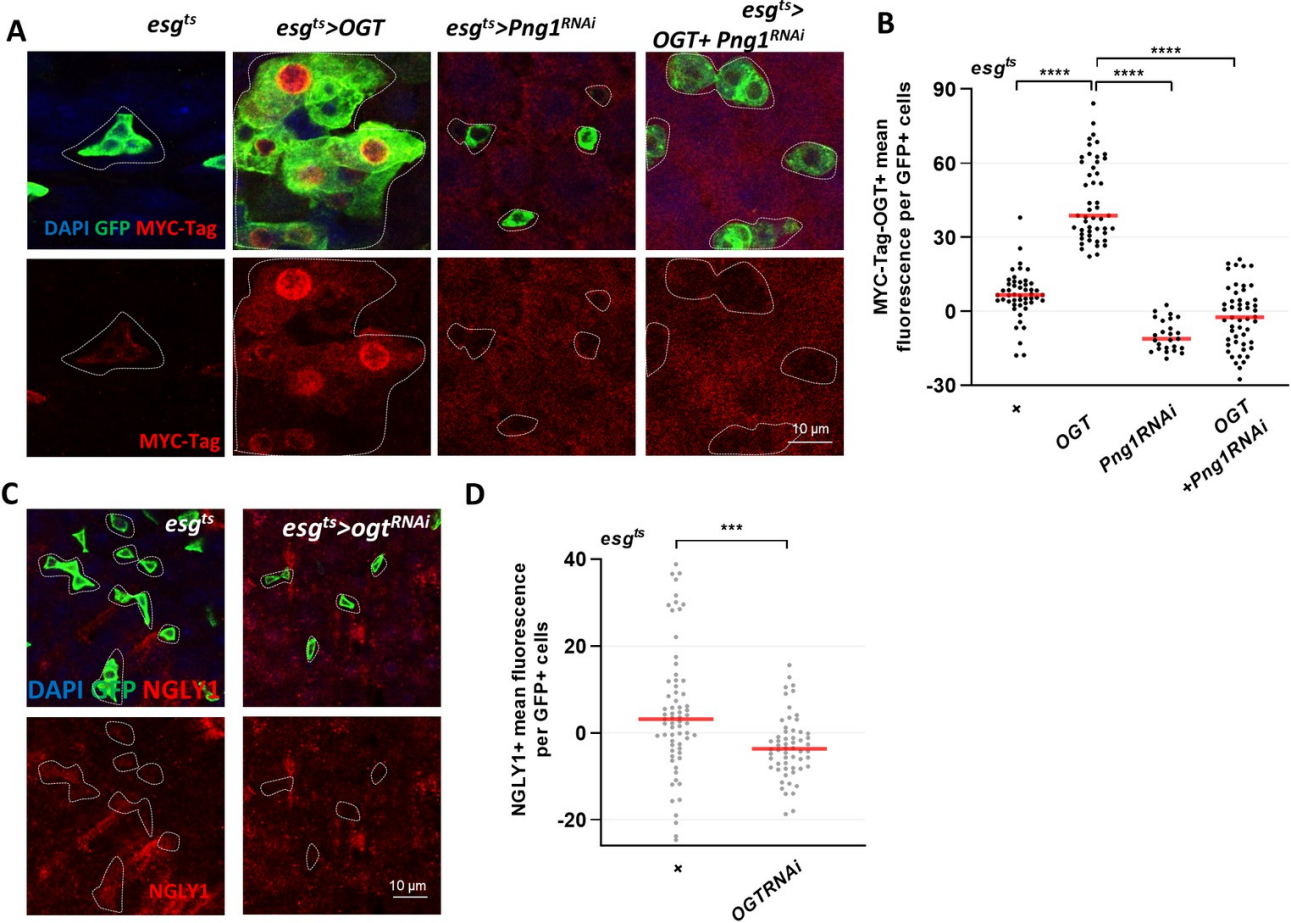
Regulation of PNG1 and OGT levels in ISCs/EBs. (A) Immunofluorescence staining of MYC-Ogt (red) in midgut of esg^ts^>Myc-Ogt, esg^ts^>Png1^RNAi^, and esg^ts^>Myc-Ogt+Png1^RNAi^ flies using an anti-Myc tag antibody. (B) Quantification of MYC mean fluorescence per esg-GFP-positive cell. p values were calculated by one-way ANOVA with correction for multiple comparisons. (C) Immunofluorescence staining of NGLY1 (red) in esg-GFP-positive cells (green) in midgut of esg^ts^ and esg^ts^>Ogt^RNAi^ flies. (D) Quantification of NGLY1 mean fluorescence per esg-GFP-positive cell from the indicated genotype. Outline indicate esg-positive cell. Data are represented as mean ± SD. ***p< 0.001. ****p< 0.0001., see S1 Table for N values.

To understand how *O-*GlcNAc might be regulated by PNG1, we assessed the association between PNG1 and OGT protein levels. We utilized a fly in which a Myc tagged OGT is overexpressed. This allows detection of the overexpressed OGT protein using an anti- Myc antibody. We examined the levels of Myc-OGT in *esg*^*ts*^*>Myc-Ogt*, *esg*^*ts*^*>Png1*^*RNAi*^, and *esg*^*ts*^*>Myc-Ogt+Png1*^*RNAi*^. We found that Myc-OGT levels significantly decreased in *esg*^*ts*^*>Myc-Ogt+Png1*^*RNAi*^ midgut compared to *esg*^*ts*^*>Myc-Ogt* midgut ISCs/EBs (Fig 3A and 3B). This indicates that PNG1 was required to maintain Myc-OGT stability. Next, we tested if OGT was required for expression of PNG1. Analysis of NGLY1 levels in OGT knockdown ISCs/EBs indicated a significant decrease as compared to control (Fig 3C and 3D). Therefore, both OGT and PNG1 are required for each other’s normal expression patterns, suggesting a mechanism by which PNG1 and OGT interaction contributes to ISC/EB proliferation.

### Induction of *O-*GlcNAcylation in ECs rescues gut dysfunction induced by PNG1 knockdown

**Fig 4:**
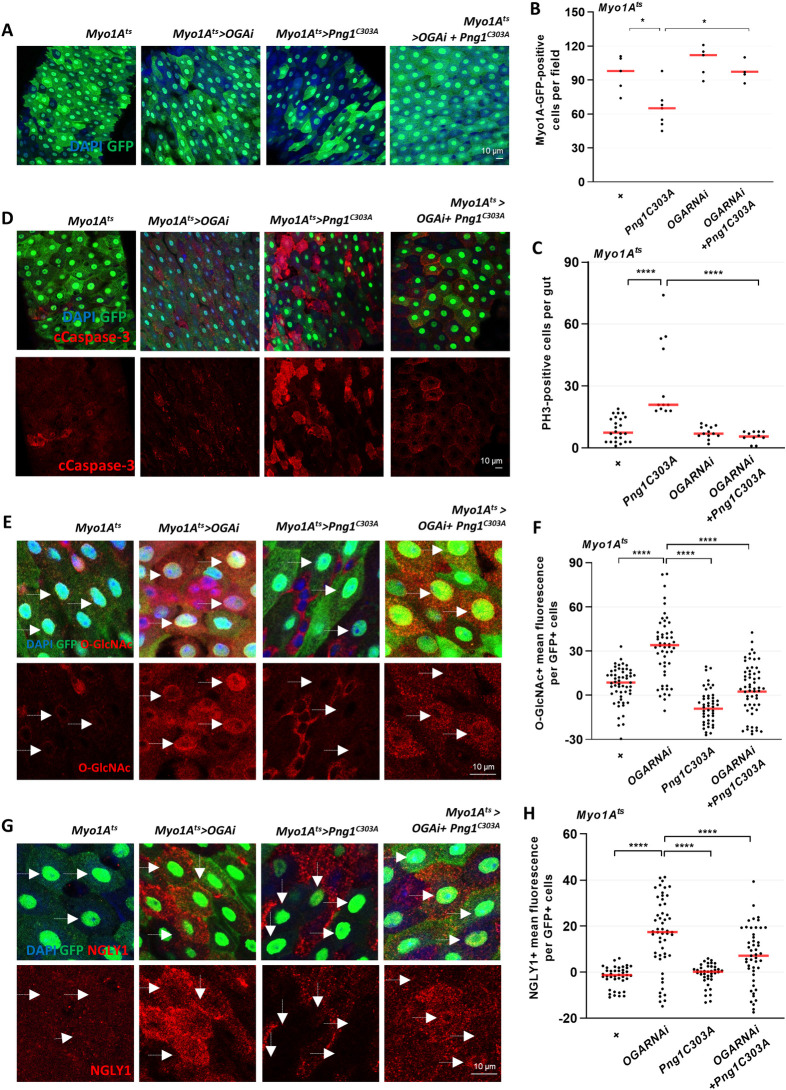
Knockdown of OGA in ECs rescues gut dysfunction in PNG1 knockdown flies. (A) After 5 days incubation at 29°C, the midgut of *Myo1A*^*ts*^*>Oga*^*RNAi*^, *Myo1A*^*ts*^*>Png1*^*C303A*^, and *Myo1A*^*ts*^*>Oga*^*RNAi*^*+Png1*^*C303A*^ flies. (B) The number of Myo1A-GFP-positive cells per field. (C) The number of PH3-positive cells in midguts from flies of the indicated genotype. (D) Immunofluorescence staining of cCaspase (red) in Myo1A-GFP-positive cells (green) in midgut of *Myo1A*^*ts*^*>Oga*^*RNAi*^, *Myo1A*^*ts*^*>Png1*^*C303A*^, and *Myo1A*^*ts*^*>Oga*^*RNAi*^*+Png1*^*C303A*^ flies. (E) Immunofluorescence staining of *O-*GlcNAc (red) in midgut of flies from the indicated genotypes. (F) Quantification of *O-*GlcNAc mean fluorescence per Myo1A-GFP-positive cell. (G) Immunofluorescence staining of NGLY1 (red) in midgut of flies from the indicated genotypes. (H) Quantification of NGLY1 mean fluorescence per Myo1A-GFP-positive cell from the indicated genotypes. White arrows indicate Myo1A-positive cell. Data are represented as mean ± SD. ****p< 0.0001., see S1 Table for N values.

Interestingly, our data indicated that ISC hyperproliferation was induced by the loss of PNG1 in ECs (Fig 1). We also observed that hyperproliferation, increased levels of PNG1, *O-*GlcNAc and ROS induced by ISC/EB-knockdown of OGA were rescued by loss of PNG1 activity (Fig 2). Thus, we wanted to explore whether PNG1-related hyperproliferation is rescued by elevating *O-*GlcNAcylation in ECs. To do this, we combined the *Png1*^*C303A*^ mutant with knockdown of OGA in ECs using an EC-specific GAL4 system. We have previously reported that ISC proliferation within the midguts of EC-specific OGA knockdown was similar to control, but ISC proliferation and cell death increased in EC-specific OGT knockdown [27]. After incubation at 29°C for 5 days, we observed a significant increase in PH3-positive cells and cCaspase-positive cells (apoptosis marker) in *Myo1A*^*ts*^*>Png1*^*C303A*^ midgut compared to controls (Fig 4A–4D), consistent with the results in Fig 1. Interestingly, there were significant decreases in both PH3-positive cell and caspase-positive cell staining in midguts from the *Myo1A*^*ts*^*>Oga*^*RNAi*^*+Png1*^*C303A*^ double-mutant flies compared to *esg*^*ts*^*>Png1*^*C303A*^ midguts (Fig 4A–4D). Thus, these data suggest that EC-specific PNG1 deficiency-induced ISC hyperproliferation and cell death can be rescued by OGA knockdown, indicating that increased *O-*GlcNAcylation is responsible for this rescue. Next, we assessed PNG1 and *O-*GlcNAc levels in these double-mutant fly midguts. PNG1 and *O-*GlcNAc levels were undetectable in *Myo1A*^*ts*^*>Png1*^C303A^ midgut GFP-positive cells (EC cells) (Fig 4E–4H). Conversely, NGLY1 and *O-*GlcNAc levels were significantly elevated in *Myo1A*^*ts*^*>Oga*^RNAi^ midgut GFP-positive cells (EC cells) (Fig 4E and 4F). Interestingly, NGLY1 and *O-*GlcNAc levels were significantly decreased in *Myo1A*^*ts*^*>Oga*^RNAi^+*Png1*^*C*303A^ double-mutant fly midgut GFP-positive cells as compared to *Myo1A*^*ts*^*>Oga*^RNAi^ (Fig 4E–4H). These findings indicate that the gut dysfunction associated with loss of PNG1 activity could be rescued by loss of OGA in ECs. Thus, PNGase and *O-*GlcNAc appear to act within the same pathway in ISC/EBs and ECs to maintain intestinal homeostasis.

### Consequences of OGT or PNG1 loss in ISCs/EBs can be rescued by modulating CncC activities

**Fig 5:**
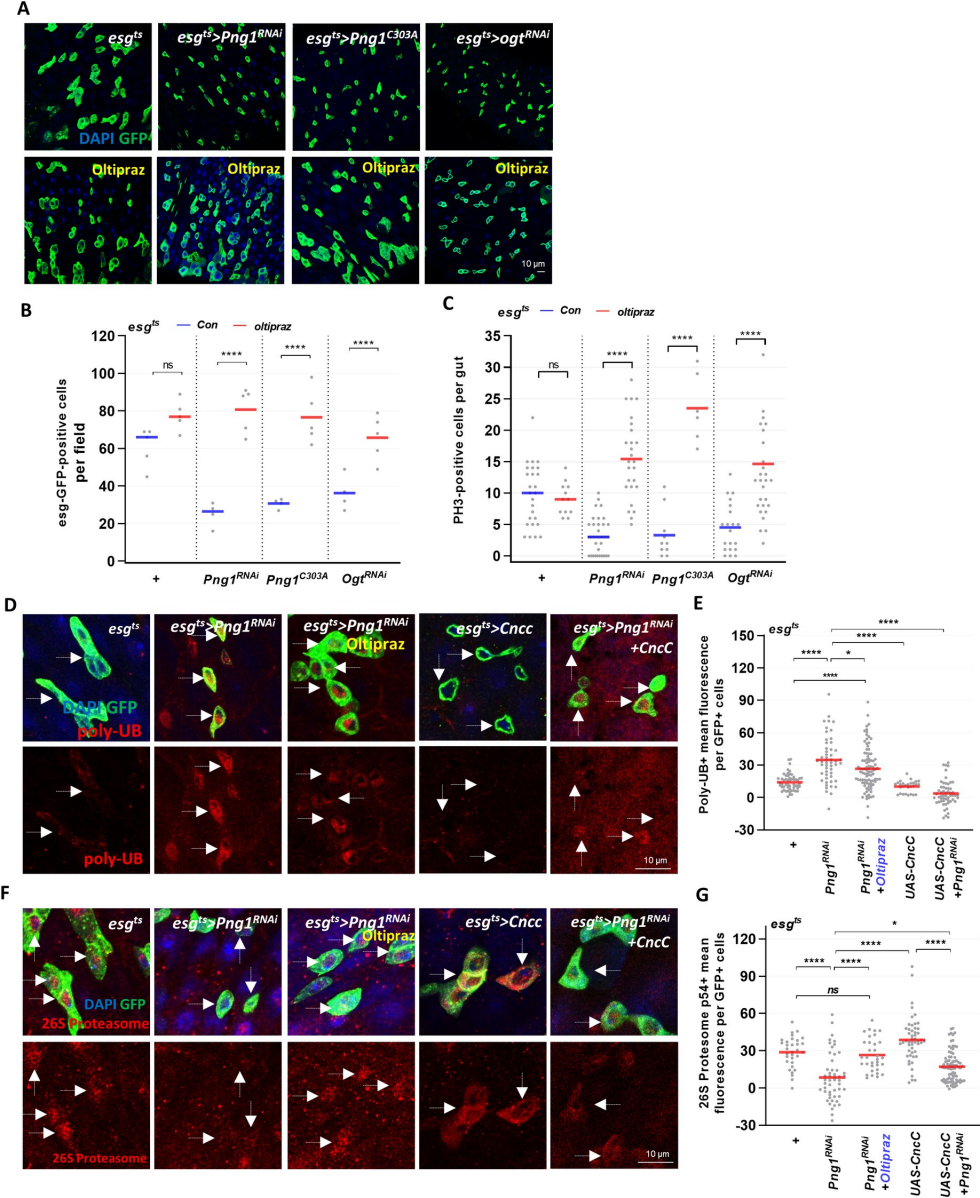
Consequences of OGT or PNG1 loss in ISCs/EBs can be rescued by modulating CncC activities. (A) The midgut of transgene without or with Oltipraz 100 uM treatment for 7 days. (B) The number of esg-GFP-positive cells per field. (C) The number of PH3-positive cells in midguts from the indicated genotype and treatment. (D) Immunofluorescence staining of poly-UB (red) in esg-GFP-positive cells (green) in midgut of flies from the indicated genotype. (E) Quantification of poly-UB mean fluorescence. (F) Immunofluorescence staining of 26S Proteasome (red) in esg-GFP-positive cells (green) in midgut of flies from the indicated genotype. (G) Quantification of 26S Proteasome mean fluorescence. White arrows indicate esg-positive cell. Outline indicates esg-positive cell. Data are represented as mean ± SD. *p< 0.05. ****p< 0.0001. n.s., not significant., see S1 Table for N values.

We have shown that both OGT and PNG1 are required for ISC proliferation and that both *O-*GlcNAc and PNG1 levels increase under stress conditions [27] (Fig 1). Cap’n’collar (CncC, the *Drosophila* Nrf2 homologue) is a transcription factor that regulates cellular redox homeostasis which has been shown to crosstalk with both *O-*GlcNAc and PNG1 [30]. Therefore, we investigated if we could rescue the proliferative in OGT knockdown and PNG1 mutation through modulation of CncC activity. To increase CncC activity, we used the chemical activator Oltipraz and examined ISC proliferation in *esg*^*ts*^*>Png1*^*RNAi*^, *esg*^*ts*^*>Png1*^*C303A*^ and *esg*^*ts*^*>Ogt*^*RNAi*^ flies treated with Oltipraz or left untreated. The number of GFP-positive cells and PH3-positive cells were not significantly changed in the Oltipraz-treated control midguts (Fig 5A–5C). Interestingly, GFP-positive and PH3-positive cells increased with Oltipraz-treatment in *esg*^*ts*^*>Png1*^*RNAi*^, *esg*^*ts*^*>Png1*^*C303A*^ and *esg*^*ts*^*>Ogt*^*RNAi*^ midguts compared to the non-treatment groups (Fig 5A–5C). Because proliferation in progenitor cells is often associated with apoptosis of differentiated cells, we examined apoptotic markers in these mutant midguts. We found that PNG1 or OGT knockdown alone did not impact apoptosis (S3 Fig). However, there was a significant increase in cCaspase-3 staining in differentiated cells after Oltipraz treatment in both PNG1 and OGT knockdown midguts compared to non-treated groups (S3 Fig). Thus, CncC activation rescued ISC proliferation and promoted apoptosis of differentiated cells. Therefore, activation of CncC can rescue the depressed ISC proliferation of the OGT and PNG1 knockdown ISCs/EBs.

We further investigated whether there was poly-ubiquitination (Poly-UB) accumulation (protein aggregation marker) and changes in proteasome activity in PNG1 knockdown associated with CncC activation. CncC is critical for activity of the proteasome and a substrate of the 26S proteasome in *Drosophila* S2 cells. In addition, the 26S proteasome is important for maintaining low levels of CncC under normal conditions but under stressed conditions CncC promotes proteasomal gene expression [31]. NGLY1 removes *N-*glycans from misfolded proteins before they can be degraded by the proteasome [17]. Our data showed that poly-UB accumulation increased in in PNG1 knockdown ISCs/EBs (Fig 5D and 5E). This increase could be rescued by CncC overexpression and chemical activation (Fig 5D and 5E). The increase in Poly-UB phenotype was associated with changes in 26S proteasome expression in the PNG1 knockdown, that was also rescued with CncC overexpression or chemical activation (Fig 5F and 5G). Therefore, the rescue of PNG1 by CncC activation was related to the regulation of protein aggregation and proteasomal degradation.

### Loss of OGT or PNG1 in ISCs/EBs can be rescued by modulating ENGase activities

**Fig 6:**
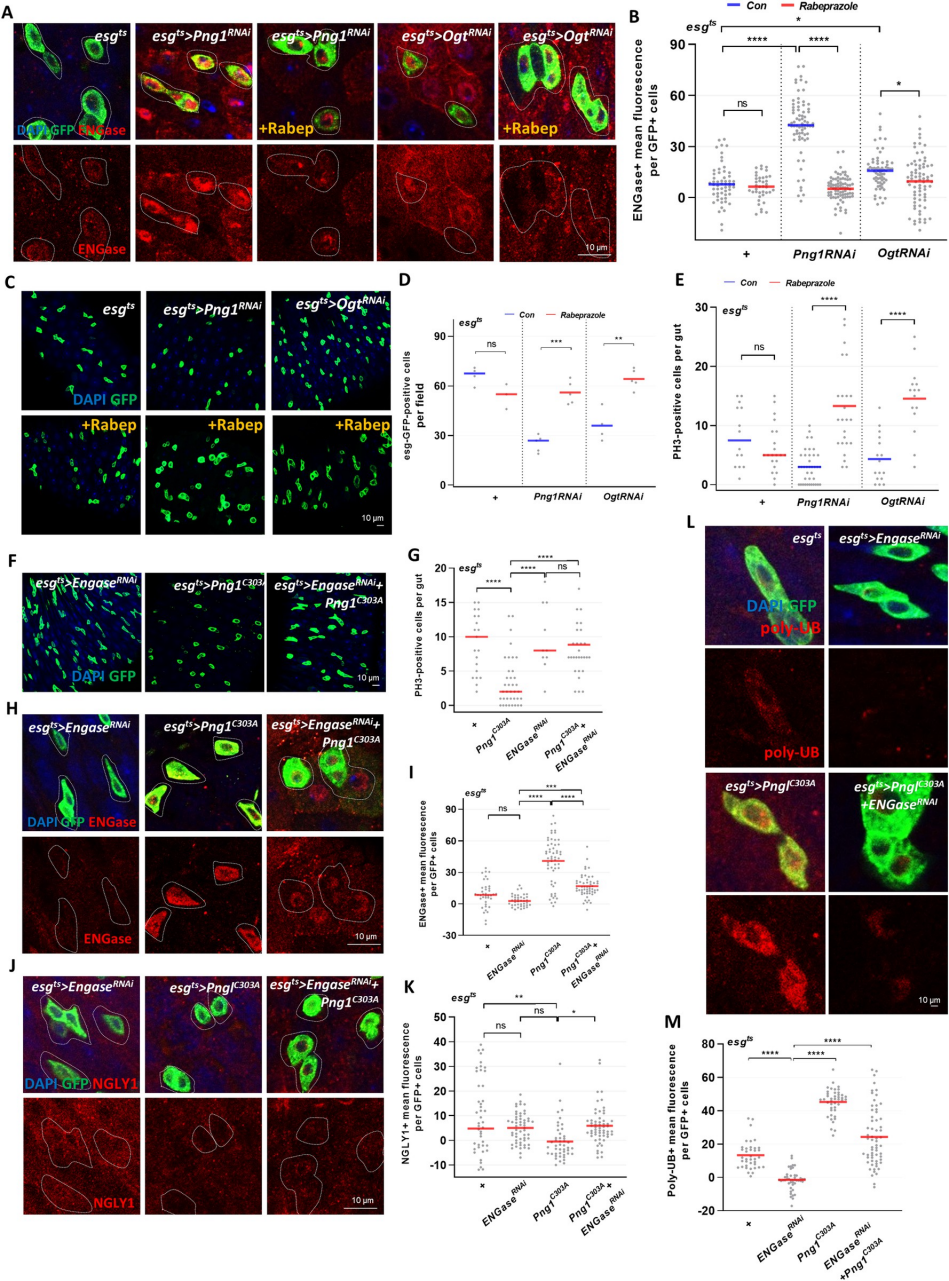
Loss of OGT or PNG1 in ISCs/EBs can be rescued by modulating ENGase activities. (A) Immunofluorescence staining of ENGase (red) in esg-GFP-positive cells (green) in midgut of flies with or without Rabeprazole 1 mM treatment for 7 days. (B) Quantification of ENGase mean fluorescence per esg-GFP-positive cell from the indicated genotype and treatment. (C) Immunofluorescence staining of esg-GFP (green) in midgut of transgene without or with Rabeprazole 1 mM treatment for 7 days. (D) The number of esg-GFP-positive cells per field. (E) The number of PH3-positive cells in midguts from the indicated genotype and treatment. (F) The midgut of *esg*^*ts*^*>ENGase*^*RNAi*^, *esg*^*ts*^*>Png1*^*C303A*^, and *esg*^*ts*^*>ENGase*^*RNAi*^ *+ Png1*^*C303A*^ flies. (G) The number of PH3-positive cells in midguts from the indicated genotype. (H) Immunofluorescence staining of ENGase (red) in esg-GFP-positive cells (green) in midgut of flies from the indicated genotype. (I) Quantification of ENGase mean fluorescence from the indicated genotypes. (J) Immunofluorescence staining of NGLY1 (red) in esg-GFP-positive cells (green) in midgut of flies from the indicated genotype. (K) Quantification of NGLY1 mean fluorescence. (L) Immunofluorescence staining of Poly-UB (red) in esg-GFP-positive cells (green) in midgut of flies from the indicated genotype. (M) Quantification of Poly-UB mean fluorescence. Outline indicates esg-positive cell. Data are represented as mean ± SD. *p< 0.05. ****p< 0.0001. n.s., not significant., see S1 Table for N values.

Endo-*β*-*N*-acetylglucosaminidase (Engase), is a cytoplasmic glycosidase that cleaves glycans on misfolded proteins. Importantly, lethality of NGLY1 deficient mice was partially rescued by additional deletion of *Engase* [17]. Thus, we wanted to define the interaction between PNG1, OGT and ENGase and test whether PNG1 and OGT mutant phenotypes could be rescued using an ENGase inhibitor (Rabeprazole; proton pump inhibitor) [32]. First, we assessed ENGase expression in ISCs/EBs-specific PNGase and OGT knockdown midguts using an anti-ENGase antibody. Interestingly, we found that ENGase levels were upregulated in *esg*^*ts*^*>Png1*^*RNAi*^ and *esg*^*ts*^*>Ogt*^*RNAi*^ midgut ISCs/EBs compared to control (Fig 6A and 6B). However, ENGase levels decreased upon Rabeprazole treatment (Fig 6A and 6B). To test whether ENGase inhibition could rescue the proliferation phenotypes of the PNG1 or OGT knockdown, we assessed GFP-positive and PH3-positive cells in *esg*^*ts*^*>Png1*^*RNAi*^ and *esg*^*ts*^*>Ogt*^*RNAi*^ treated with Rabeprazole and found significant increases in proliferation as compared to the non-treated controls (Fig 6C–6E). Consistently, cCaspase-3 staining was elevated in differentiated cells from the Rabeprazole treated PNG1 and OGT knockdown midguts compared to non-treated groups (S3 Fig). To look more specifically at ERAD and ER stress and tease out if extracellular *N-*Glycosylation might be playing a role, we assessed ISC proliferation and levels of ENGase using double combination PNG1 mutant and ENGase knockdown flies. Our data showed that the number of GFP-positive and PH3-positive cells of *esg*^*ts*^*>ENGase*^*RNAi*^ were similar to control, and ENGase was undetectable in ENGase^RNAi^ ISCs/EBs (Fig 6F, 6G and 6H). Interestingly, PNG1 levels did not change in ENGase^RNAi^ ISCs/EBs compared to control (Fig 6J). We found that ISC proliferation and PNG1 levels in PNG1 mutant flies were rescued by ENGase knockdown (Fig 6F, 6G, 6J and 6K). The increased ENGase levels were rescued by ENGase knockdown in ISCs/EBs (Fig 6H and 6I). We next investigated protein aggregation and assessed poly-ubiquitination accumulation in PNG1 mutant ISCs/EBs. PNG1 knockdown alone increased poly-ubiquitination accumulation, however, this was rescued by ENGase knockdown in ISCs/EBs (Fig 6L and 6M). Thus, our data indicated that protein aggregation and ENGase activity was likely associated with stem cell proliferation in loss of PNG1. Moreover, PNG1-related gut homeostasis is controlled through ENGase activity. Therefore, our data indicates a pathway in which PNG1 and OGT inhibit ENGase, with ENGase having an inhibitory impact on ISC proliferation.

### EC-specific OGT or PNG1 knockdown-induced ISC proliferation was rescued by CncC or ENGase activities

**Fig 7:**
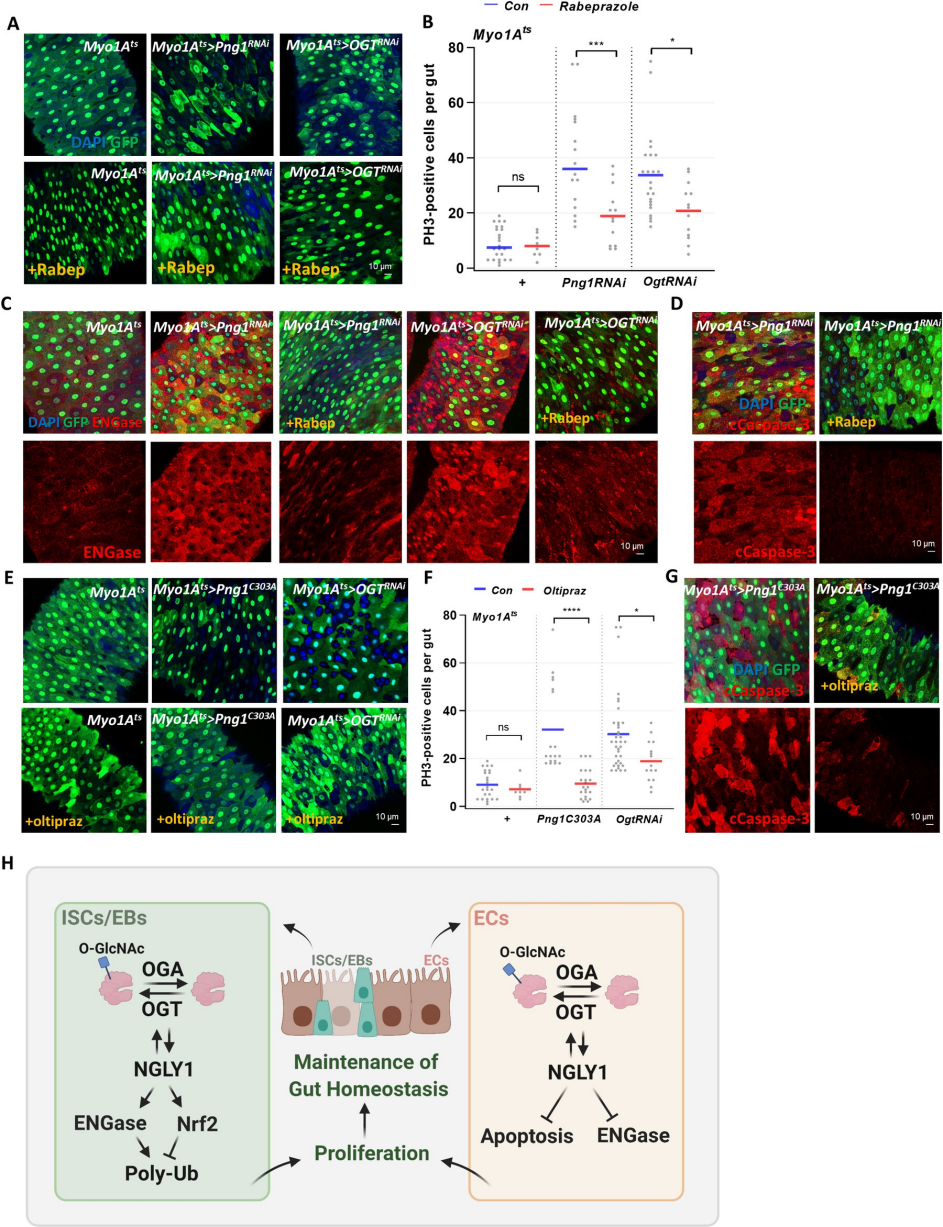
Modulating CncC or ENGase activity rescues EC-specific OGT or PNG1 knockdown phenotypes. (A) The midgut of transgene without or with Rabeprazole 1 mM treatment for 5 days. (B) The number of PH3-positive cells in midguts from the indicated genotype and treatment group. (C) Immunofluorescence staining of ENGase (red) in Myo1A-GFP-positive cells (green) in midgut of flies of the indicated genotype with or without Rabeprazole. (D) Immunofluorescence staining of cCaspase (red) in Myo1A-GFP-positive cells (green) in midgut of flies from the indicated genotype with or without Rabeprazole. (E) Immunofluorescence staining of Myo1A-GFP (green) in midgut of transgene without or with Oltipraz 100 uM treatment for 5 days. (F) The number of PH3-positive cells in midguts from flies of the indicated genotpe and treatment. (G) Immunofluorescence staining of cCaspase (red) in Myo1A-GFP-positive cells (green) in midgut of flies from the indicated genotype with or without Oltipraz. (H) *O-*GlcNAc and PNG1 cooperate to influence intestinal homeostasis in *Drosophila* which can be modulated by Nrf2 and ENGase activity. Data are represented as mean ± SD. *p< 0.05. ***p< 0.001. ****p< 0.0001. n.s., not significant., see S1 Table for N values.

Our data indicated ISC-specific OGT or PNG1 knockdown induced ENGase expression and decreased ISC proliferation, which could be rescued by ENGase inhibition or CncC activation (Figs 5 and 6). Next, we assessed whether ENGase inhibition or CncC activation could also rescue the phenotypes of PNG1 or OGT knockdown in differentiated cells. As shown Fig 7A and 7B, ISC hyperproliferation of *Myo1A*^*ts*^*>Png1*^*RNAi*^ and *Myo1A*^*ts*^*>Ogt*^*RNAi*^ was rescued by Rabeprazole treatment. Interestingly, ENGase expression was increased in *Myo1A*^*ts*^*>Png1*^*RNAi*^ and *Myo1A*^*ts*^*>Ogt*^*RNAi*^ midgut and was decreased in the Rabeprazole treated compared to non-treated groups (Fig 7C). Further, EC-specific PNG1 knockdown-induced cell death was decreased in *Myo1A*^*ts*^*>Png1*^*RNAi*^ with Rabeprazole (Fig 7D). Thus, in ECs, loss of OGT or PNG1 induced ISC proliferation, ENGase, and cell death was rescued by inhibition of ENGase. Next, we tested whether CncC activation could rescue hyperproliferation of EC-specific PNG1 or OGT knockdown. As shown Fig 7E and 7F, ISC hyperproliferation of *Myo1A*^*ts*^*>Png1*^*RNAi*^ and *Myo1A*^*ts*^*>Ogt*^*RNAi*^ was rescued by Oltipraz treatment. Moreover, EC-specific PNG1 knockdown-induced cell death was decreased in *Myo1A*^*ts*^*>Png1*^*RNAi*^ with Oltipraz (Fig 7G). Thus, loss of OGT or PNG1 induced ISC proliferation and cell death was rescued by CncC activation. Therefore, ENGase and CncC regulate OGT and PNG1 to maintain tissue homeostasis. A model depicting the roles of PNG1 and OGT in ISCs/EBs and ECs resulting in altered proliferation and apoptosis is shown in Fig 7H. Thus, *O*-GlcNAcylation and PNG1 have key roles in both progenitor (ISCs/EBs) and differentiated cells (ECs) contributing to tissue homeostasis. PNG1 regulates stem cell proliferation and is elevated in OGA knockdown midgut ISCs/EBs. PNG1 knockdown was rescued by *O*-GlcNacylation-induced dysplasia. PNG1 and OGT regulate each other’s levels in ISCs/EBs. PNG1 knockdown-induced gut dysfunction was rescued by induction of *O*-GlcNacylation in ECs. OGT and PNG1 knockdown in ISCs/EBs induced ENGase, resulting in reduced proliferation. In ECs, OGT and PNG1 knockdown induced ENGase and cell death further promoting hyperproliferation of ISC/EBs. Phenotypes of OGT and PNG1 knockdown in ISCs/EBs or ECs were rescued by modulation of ENGase or CncC level (Fig 7H). Taken together, OGT and PNG1 are important factors regulating ISC proliferation, and cell death in *Drosophila* whose effects can be mitigated by modulating CncC and ENGase.

## Discussion

Intestinal stem cells regulate tissue homeostasis by balancing self-renewal, proliferation, and differentiation all of which are supported by elevated flux through the HBP [26]. Both *N-*linked glycosylation and intracellular *O-*GlcNAc modifications are regulated by the HBP pathway in a nutrient-sensing manner [2,3]. However, how NGLY1 is utilized to control stem cell homeostasis and differentiation in cells remains largely unknown. This is a critical question as patients with NGLY1-deficiency display global developmental delay, movement disorder and growth retardation [14,15]. Elevation of NGLY1 was observed in patients’ tumor samples, suggesting a function in oncogenic signaling [20]. In *Drosophila*, PNG1 mutants had severe developmental defects and reduced viability, with the surviving adults frequently sterile [16]. Here, we identified a pathway by which PNG1 regulates ISC homeostasis *in vivo*. In this study, we show that PNG1 levels increased in ISC/EBs concomitant with *O-*GlcNAc. This interaction between PNG1 and *O-*GlcNAcylation is critical for maintaining normal ISC proliferation and differentiation. Thus, through their mutual regulation, OGT and PNG1 have key roles in both progenitor (ISCs/EBs) and differentiated cells (ECs) contributing to tissue homeostasis. Previous reports indicated that PNG1 null larvae have specific developmental abnormalities in their midgut that contributes to their lethality [28]. Further, intestinal inflammation in Crohn’s disease is associated with increased *O-*GlcNAc modification [29]. Our previous study also showed that increased *O-*GlcNAc promotes gut dysplasia through regulation of DNA damage [27]. Thus, PNG1 or *O-*GlcNAc might still be associated with gut dysfunction in a disease context.

The regulation of *O-*GlcNAc by PNG1 and the interaction between PNG1 and *O-*GlcNAc has been implicated previously. In fact, GlcNAc supplementation partially rescued lethality associated with PNG1 knockdown [33]. Although the mechanism by which GlcNAc supplementation rescued these mutant flies has not been fully worked out, Gfat1 transcript levels were downregulated in PNG1 knockdown flies [33]. Gfat1 is the enzyme that controls the rate limiting step in the HBP to produce UDP-GlcNAc. Thus, PNG1 through regulation of Gfat1 could impact levels of UDP-GlcNAc and ultimately *O-*GlcNAc [26,33]. Additionally, it has been hypothesized that the loss of PNG1 could increase the presence of intracellular *N-*GlcNAc modification, potentially interfering with *O-*GlcNAc mediated signaling [34]. Therefore, alterations in UDP-GlcNAc levels or presence of intracellular *N-*GlcNAc upon PNG1-deficiency can interact with *O-*GlcNAc to regulate stem cell homeostasis.

Previous reports have shown that Nrf1 undergoes NGLY1-mediated deglycosylation, followed by proteolytic cleavage and translocation into the nucleus as an active transcription factor [13]. Loss of NGLY1 caused Nrf1 dysfunction, as evidenced by an enrichment of deregulated genes encoding proteasome components and proteins involved in oxidation reduction [33]. Proteasome activity can induce an apoptotic cascade that leads to growth arrest and, subsequently, cell death. Our data indicated that PNG1 or OGT knockdown suppressed ISC proliferation, which was rescued by Oltipraz (CncC activation) treatment in ISCs/EBs (Fig 5). Furthermore, there was increased apoptosis in PNG1 or OGT knockdown with treatment compared to non-treated groups (S3 Fig). Interestingly, we found *O-*GlcNAc-induced intestinal dysplasia was rescued by knockdown of PNG1 in ISCs/EBs through regulation of ROS levels (Fig 2G). Similarly, increases in global *O-*GlcNAcylation in embryos of diabetic mice caused an overproduction of ROS and subsequent oxidative and ER stress. It is known that activation of SKN-1A/Nrf1 also requires deglycosylation by PNG-1/NGLY1 in *C*. *elegans* [18]. Further, SKN-1 is *O-*GlcNAc modified [35] and translocates to the nucleus in *ogt*-1(ok430)-null worms [36]. Together, these studies all suggest conserved functional connections between *O-*GlcNAc and Nrf family transcription factors [37]. Here, we also showed EC-specific OGT or PNG1 knockdown-induced hyperproliferation and cell death was decreased by CncC activation (Fig 7). This data indicated OGT or PNG1 can be regulated by CncC activity in ISCs/EBs and ECs. CncC has high activity within ISCs/EBs of unstressed as well young ISCs and quiescent ISCs but decreases with age and damage [38,39]. These data indicated that CncC acts to properly balance between signaling and damage responses necessary for tissue homeostasis [38]. We found that CncC activation increased ISC proliferation in ISCs/EBs and decreased ISC proliferation in ECs of OGT or PNG1 knockdown contributing towards tissue homeostasis. Another study showed that inhibition of NGLY1 resulted in Nrf1 being misprocessed, mislocated, and inactive, thus indicating that functional NGLY1 is essential for Nrf1 processing, nuclear translocation, and transcription factor activity [40]. Therefore, our data suggests that PNG1 and OGT modulated by CncC activation contribute to ISC proliferation and ultimately regulating tissue homeostasis. Nrf2 activation was able to rescue the developmental growth of NGLY1 deficiency in worm and fly models [41]. In cancer-initiating cells, ER stress-dependent (ROS-independent) CncC induction is an event necessary to maintain stemness [42]. Our data showed that PNG1 knockdown-induced Poly-UB accumulation and 26S proteasome expression that was rescued by CncC overexpression and chemical activation (Fig 5). Through functioning as a sensor of cytosolic proteasome activity and an activator of aggresomal formation, Nrf2 alleviates cell damages caused by proteasomal stress [43]. Expression of proteasome subunit genes and mitophagy-related genes were broadly enhanced after sulforaphane (Keap1 inhibitor) treatment and pharmacologically induction of Nrf2 promotes mitophagy and ameliorates mitochondrial defect in *Ngly1*^*−/−*^ cells [42]. Thus, we believe that the sensitized background of the OGT or PNG1 mutant provides an environment where CncC activation promotes proliferation to the normal level through regulation of proteasome activity and protein aggregation.

In our previously published paper [27], we showed OGT overexpression and OGA knockdown in ISCs/EBs both increased *O-*GlcNAc levels and induced hyperproliferation of the stem cells, whereas OGT knockdown decreased proliferation. However, in differentiated ECs, OGT overexpression and OGA knockdown phenotypes were similar to the normal gut, whereas OGT knockdown elevated proliferation and cell death. In general, EC death promotes proliferation in order to maintain gut homeostasis [24]. Here, NGLY1 knockdown in ISCs/EBs decreased proliferation and clone size (Figs 1 and S2) but NGLY1 knockdown in ECs induced hyperproliferation and cell death and importantly decreased *O-*GlcNAc levels. Thus, the phenotypes of OGT and NGLY1 were similar, demonstrating that maintenance of OGT and NGLY1 protein expression is highly interdependent for the maintenance of tissue homeostasis. It is interesting that the progenitor and differentiated cell types within the gut respond differently to changes in *O-*GlcNAc. It is possible that a certain level of *O-*GlcNAcylation is needed to maintain stem cells and promote proliferation and self-renewal, however, differentiated cells that do not have the same energy and growth requirements are not as reliant on high levels of *O-*GlcNAc. On the other hand, both ISC/EBs and ECs require some level of *O-*GlcNAc and without OGT there is decreased proliferation in progenitor cells and increased cell death of ECs. There are a few possibilities how NGLY1 and OGT can collaboratively work, however, it is unlikely that they share protein targets. First, a previous publication showed that additional deletion of ENGase, another N-deglycosylating enzyme that leaves a single GlcNAc residue, alleviates some of the lethality of Ngly1-deficient mice [17]. Thus, it is possible with the accumulation of aggregation prone intracellular N-GlcNacylated proteins, there is disruption of normal *O-*GlcNac signaling. Our data also showed increased protein aggregation in OGT or NLGY1 knockdown that was rescued by ENGase knockdown (Fig 6). In addition, MYC-OGT protein levels in OGT overexpression fly guts were decreased by PNG1 knockdown (Fig 3). It is possible that loss of NGLY1 disrupts normal OGT degradation and thus impacts levels global of *O-*GlcNAcylation.

In this study we have shown that ENGase levels increased in PNG1 or OGT knockdown ISCs/EBs and ECs (Figs 6 and 7). PNGase is involved in the process of endoplasmic reticulum associated degradation (ERAD), acting as a deglycosylating enzyme that cleaves *N-*glycans attached to ERAD substrates [12]. The small molecule ENGase inhibitors have potential to treat pathogenesis associated with NGLY1 deficiency [44]. Rabeprazole, a proton pump inhibitor, was identified as a potential ENGase inhibitor [44]. We demonstrated that the consequences of knockdown of OGT or PNG1 on ISC proliferation and ENGase activity was rescued by Rabeprazole treatment in ISCs/EBs or ECs (Figs 6 and 7). Our data showed that cell death was elevated in ISCs/EBs-specific PNG1/OGT knockdown with Rabeprazole treatment compared to non-treated groups concomitant with an increase in ISC proliferation (S4 Fig). On the other hand, cell death decreased in EC-specific PNG1 knockdown treated with Rabeprazole resulting in a decrease in ISC proliferation (Fig 7). It is known that loss of PNG1 function in cells can cause the accumulation of aberrant proteins in the cytosol and the interruption of ERAD. Further, downregulation of ER stress-related genes has been reported in B-cell-specific OGT mutant mice [45]. The protective effects of *O-*GlcNAc are not limited to mitochondrial function but also rescue injury caused by ER stress [46]. Therefore, NGLY1/OGT seems to be functionally associated with the ERAD machinery [17]. More recently, using a model ERAD substrate, it was reported that the ablation of *Ngly1* causes a disruption in the ERAD process in mouse embryonic fibroblast (MEF) cells [47]. Moreover, lethality of mice bearing a knockout of the *Ngly1*-gene was partially rescued by the additional deletion of the *Engase* gene [17]. Interestingly, we showed that OGA knockdown rescued ENGase levels of PNG1 knockdown ISCs/EBs (S4 Fig). Hence, these findings suggest that there is a correlation between OGT/PNG1 and ENGase contributing to tissue maintenance.

Taken together, our findings implicate *O-*GlcNAc and PNG1 as key regulators of tissue maintenance. PNG1 can impact stem cell homeostasis through regulation of *O-*GlcNAc both in ISCs/EBs or ECs. Of significance is the finding that PNG1 and OGT phenotypes are rescued by modulating CncC and ENGase activity in ISCs/EBs or ECs. Thus, our findings reveal that nutrient-driven glycosylation contribute towards control of ISC and progenitor cell proliferation and EC cell death via regulation of CncC and ENGase. Our study provides a platform for future designs of interventions in which changes in *O-*GlcNAc can be utilized as a therapeutic for stem-cell-derived diseases like cancer. We also present a molecular mechanism and unexpected pathway that can be targeted for treating NGLY1-deificient patients.

### Materials availability

This study did not generate new unique reagents.

## Materials and methods

### Drosophila stocks, culture, and husbandry

Fly stocks were maintained at 25°C on standard food under a ∼12 h/12 h light/dark cycle. Food consisted of 15.8g yeast, 9g soy flour, 5.2g agar, 67g cornmeal, and 0.5% propionic acid. To avoid larval overpopulation, <30 adult flies per vial were transferred to new food vials every 2–3 days.

The following stocks were used in this study: esg-Gal4,tub-Gal80ts,UAS-GFP/CyO (esg^ts^>GFP (gift from Bruce Edgar) [48]; FRT82B, tub-Gal80/TM6B (gift from Bruce Edgar) [49]; Myo1A-Gal4,tub-Gal80ts,UAS-GFP/CyO (Myo1A^ts^> GFP) (gift from Bruce Edgar) [48]; UAS-CncC (gift from Dirk Bohmann) [50]; UAS-Png1WT; UAS-Png1C303A; Png1ex18; Png1ex14 (gift from Hamed Jafar-Nejad) [28]; UAS-OGARNAi (VDRC, #106670); UAS-OGARNAi (VDRC, #41822); UAS-OGTRNAi (VDRC, #18610); UAS-OGTRNAi (VDRC, #18611); UAS-Png1RNAi (VDRC, #103607); w^1118^ (BDSC, #3605); UAS-Png1RNAi (BDSC, #54853); UAS-ENGase RNAi (BDSC, #64609).

The UAS-OGT line that contains Myc epitope tag in the N terminus (UAS-Myc-OGT) was made by P-element-mediated transformation and OGAdel.1 mutant was generated by standard P-element excision [51].

For transgene expression at specific developmental stages, the Gal80ts technique was used. The flies were set up and maintained at 22°C until adulthood. After maintaining the flies at 29°C, the midguts were dissected and analyzed.

#### Paraquat feeding assay

Flies were treated with 10 mM paraquat for 20–22 h at 29°C. After feeding, the midguts were dissected and analyzed.

#### Oltipraz feeding assay

Two-day-old flies were fed 100 uM Oltipraz (Selleckchem, #S7864) or DMSO only mixed in standard food for 7 days or 5 days at 29°C. Flies were transferred to new Oltipraz-containing food vials every 2 days.

#### Rabeprazole feeding assay

Two-day-old flies were fed 1 mM Rabeprazole (Selleckchem, #S4845) or DMSO only mixed in standard food for 7 days or 5 days at 29°C. Flies were transferred to new Rabeprazole -containing food vials every 2 days.

#### DHE assay

Fly midguts were dissected in Schneider’s medium (HyClone). After incubation in 30 μM DHE (Invitrogen) for 7 min in the dark at room temperature, midguts were washed three times and mounted. Images were captured immediately with a Zeiss LSM700 confocal microscope.

#### MARCM

For MARCM experiments, flies were maintained at 23°C until 3–5 days after eclosion, heat-shocked at 37°C for 60 min, and then maintained back at 23°C before dissection and markers were analyzed at 7 day after induction.

#### Immunochemistry

Intact adult guts were dissected, fixed at room temperature for 1 h in 4% para-formaldehyde (PFA), washed with PBST [0.1% Tween 20 in phosphate-buffered saline (PBS)], and incubated overnight with primary antibody at 4°C. The primary antibody used in this study include rabbit phospho-Histone H3 (Ser10) (Millpore, Cat# 06–570, 1:500 dilution); mouse anti-Green fluorescent protein (GFP) (DSHB, Cat# DSHB-GFP-4C9, 1:100 dilution); rabbit anti-GFP (Thermo Fisher Scientific, Cat# A-11122, 1:500 dilution); mouse anti-*O-*linked *N-*acetylglucosamine (*O-*GlcNAc) (HGAC85) (Thermo Fisher Scientific, Cat# MA1-076, 1:50 dilution); rabbit anti-cCaspase-3 (Cell signaling, Cat#9661S, 1:100 dilution); rabbit anti-ENGase (biorbyt, Cat# orb183396, 1:100 dilution); rabbit anti-IgG (proteintech, Cat# 14678-1-AP, 1:100 dilution); rabbit anti-ENGase (ATLAS ANTIBODIES, Cat# HPA021551, 1:50 dilution); mouse anti-26S Proteasome p54 (28) (Santa Cruz, Cat# sc-65748, 1:50 dilution); mouse mono- and polyubiquitinylated conjugates recombinant (Enzo, Cat# ABS840-0100, 1:100 dilution); rabbit NGLY1 (Thermo Fisher Scientific, Cat# PA5-57836, 1:100 dilution); mouse anti-Myc tag (abcam, Cat# ab18185, 1:50 dilution). The samples were then incubated for 2 h with secondary antibodies at 25°C. The secondary antibody used in this study include Goat anti-Rabbit Antibody Alexa Fluor 635; Goat anti-Mouse Antibody Alexa Fluor 568; Goat anti-Mouse Antibody Alexa Fluor 488; Goat anti-Rabbit Antibody, Alexa Fluor 568; and Goat anti-Rabbit Antibody, Alexa Fluor 488 (Thermo Fisher Scientific, 1:300 dilution). After washing in PBST, slides were mounted with Vectashield and analyzed using a Zeiss LSM 700 system.

#### Quantitative of PH3 positive cell

To quantitatively analyze PH3-positive cells, the number of PH3-positive cells in the whole gut was counted. N represents the number of guts.

#### Quantitative of Delta- or GFP-positive cell

To quantitatively analyze Delta- or GFP-positive cells, the number of Delta- or GFP-positive cells in the field was counted. N represents the number of guts.

#### Measurement of O-GlcNAc, NGLY1, Myc-OGT, DHE, 26S proteasome, poly-UB, and ENGase fluorescence in ISC

The fluorescence images of *O-*GlcNAc, NGLY1, Myc-OGT, DHE, 26S proteasome, poly-UB, and ENGase staining were captured at the same exposure time in each experiment and was measured by quantifying the level of fluorescence (IHC staining) in individual ISCs normalized to nearby background in FIJI (ImageJ) software. The mean fluorescence was analyzed after exclusion of the mean of the background region (from two spots, excluding the nuclear portion in the posterior midgut), with background fluorescence set to 0. At least 10 ISCs were quantified in each image, and >10 images (>1 image per fly) were used to calculate the average intensity of fluorescence in ISCs of each fly. N represents the number of guts. n represents the number of cells.

### Statistical analysis

Data representation and statistical analysis were performed using GraphPad Prism software. Statistical analysis was performed using a *t*-test and multiple comparisons were performed with a One-Way ANOVA. All experiments were replicated independently 2–3 times. N represents the number of guts. n represents the number of cells.

## Supporting information

S1 Fig ISC proliferation and cell death induce in EC-specific OGT or Png1 knockdown midgut. (A), Immunofluorescence staining of Delta (red) in Myo1A-GFP-positive cells (green) in midgut of flies. (B) The number of Delta-positive cells or Myo1A-GFP-positive cells per field. (C) The percentage of Delta-positive cells vs. Myo1A-GFP-positive cells per field. Data are represented as mean ± SD. **p< 0.01. ***p< 0.001. ****p< 0.0001. n.s., not significant., see S1 Table for N values.
(TIF)

S2 Fig Clone size, *O*-GlcNAc, and NGLY1 decreased in Png1 or OGT mutant midgut used MARCM clonal system. (A) In the midgut of FRT82, *Png1*^*ex18*^; FRT82B, and *sxc*^*7*^; FRT82B flies. (B) The number of clones per field in midguts from the indicated genotype. (C) The number of cells per clone in midguts from the indicated genotype. (D) Immunofluorescence staining to analyze GFP (green) and *O*-GlcNAc (red) in midguts flies. (E) Immunofluorescence staining to analyze GFP (green) and NGLY1 (red) in midguts flies. White arrows indicate GFP-positive cell. Data are represented as mean ± SD. ***p< 0.001. ****p< 0.0001., see S1 Table for N values.
(TIF)

S3 Fig cCaspase-3 positive cell increased in Png1 knockdown and OGT knockdown midguts with Rabeprazole treatment. Immunofluorescence staining images of cCaspase-3 (red) in esg-GFP-positive cells (green) in midgut of Oltipraz or treatment in *esg*^*ts*^, *esg*^*ts*^*>Png1*^*C303A*^, and *esg*^*ts*^*>Ogt*^*RNAi*^ flies.
(TIF)

S4 Fig OGA knockdown rescues ENGase levels in PNG1 knockdown in ISCs/EBs. Immunofluorescence staining images of ENGase (red) in esg-GFP-positive cells (green) in midgut of *esg*^*ts*^, *esg*^*ts*^*>Oga*^*RNAi*^, *esg*^*ts*^*>Png1*^*C303A*^ and esg^ts^>Oga^RNAi^+Png1^C303A^ flies.
(TIF)

S1 Table N value of Figs. (DOCX)
