# Supplementary figures and images for: Cytosolic O-GlcNAcylation and PNG1 maintain Drosophila gut homeostasis by regulating proliferation and apoptosis
Source: PLoS Genet. 2022 Mar 16;18(3):e1010128. doi: 10.1371/journal.pgen.1010128 (PMC8959174; doi:10.1371/journal.pgen.1010128)

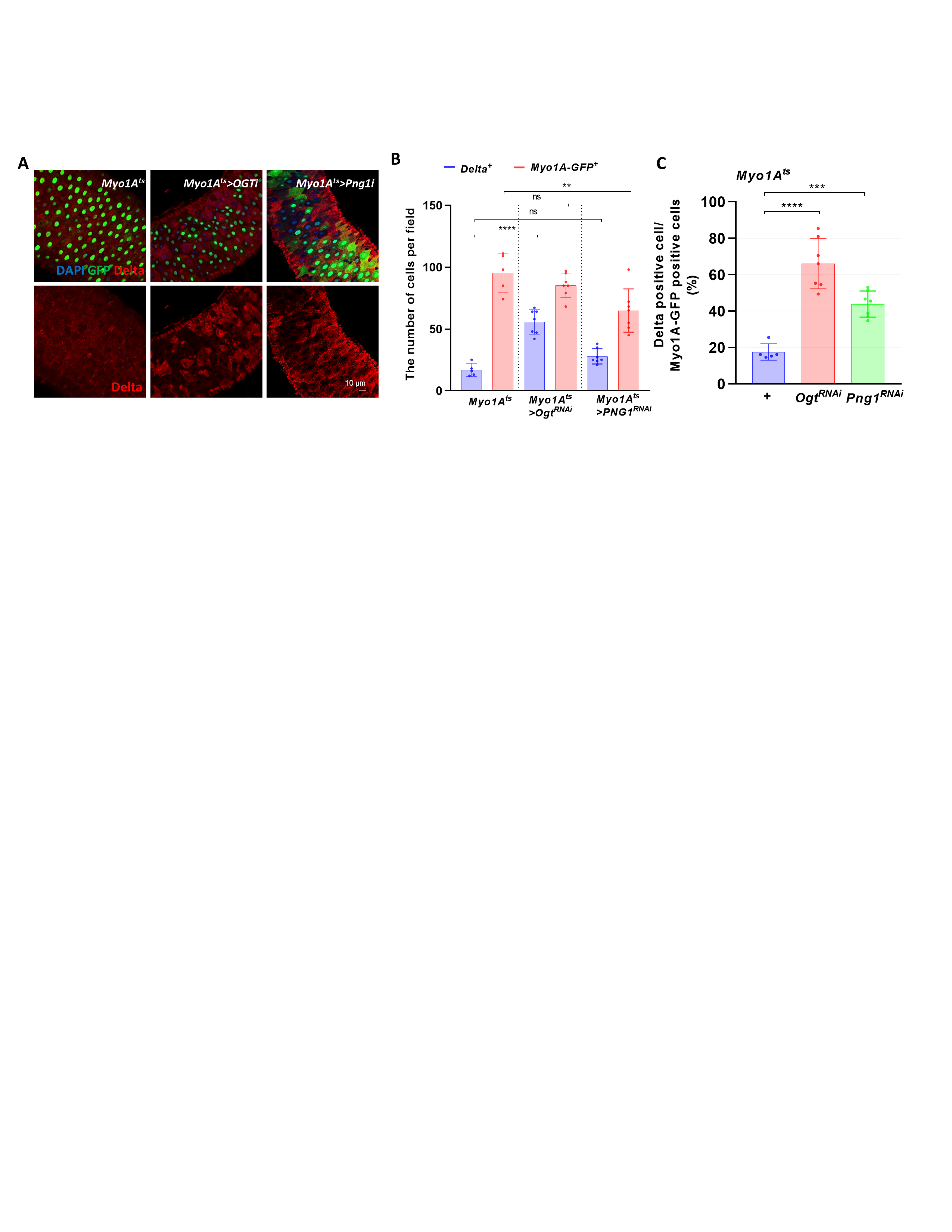

Supplement: S1 Fig — (A), Immunofluorescence staining of Delta (red) in Myo1A-GFP-positive cells (green) in midgut of flies. (B) The number of Delta-positive cells or Myo1A-GFP-positive cells per field. (C) The percentage of Delta-positive cells vs. Myo1A-GFP-positive cells per field. Data are represented as mean ± SD. **p< 0.01. ***p< 0.001. ****p< 0.0001. n.s., not significant., see S1 Table for N values. (TIF) [file pgen.1010128.s001.tif]

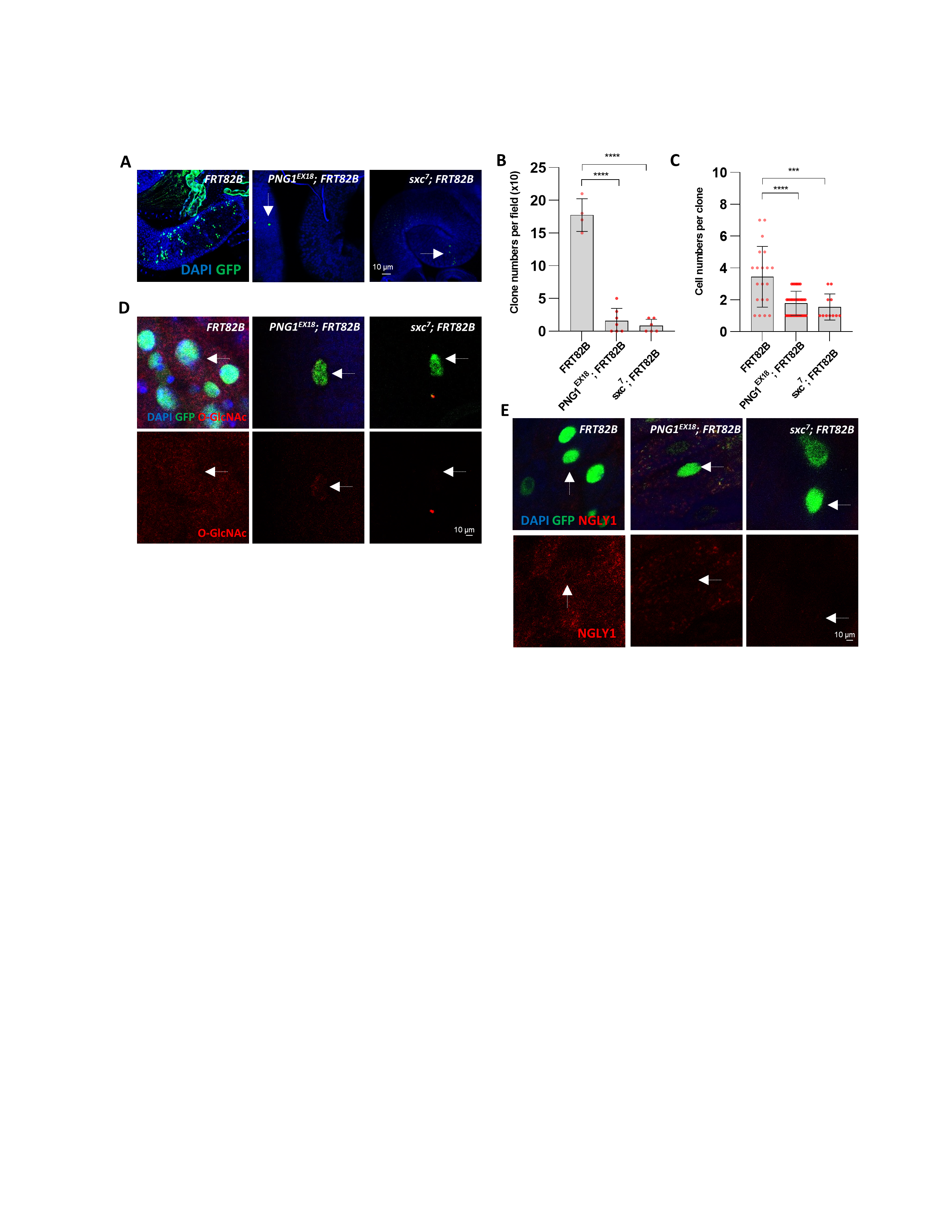

Supplement: S2 Fig — (A) In the midgut of FRT82, Png1ex18; FRT82B, and sxc7; FRT82B flies. (B) The number of clones per field in midguts from the indicated genotype. (C) The number of cells per clone in midguts from the indicated genotype. (D) Immunofluorescence staining to analyze GFP (green) and O-GlcNAc (red) in midguts flies. (E) Immunofluorescence staining to analyze GFP (green) and NGLY1 (red) in midguts flies. White arrows indicate GFP-positive cell. Data are represented as mean ± SD. ***p< 0.001. ****p< 0.0001., see S1 Table for N values. (TIF) [file pgen.1010128.s002.tif]

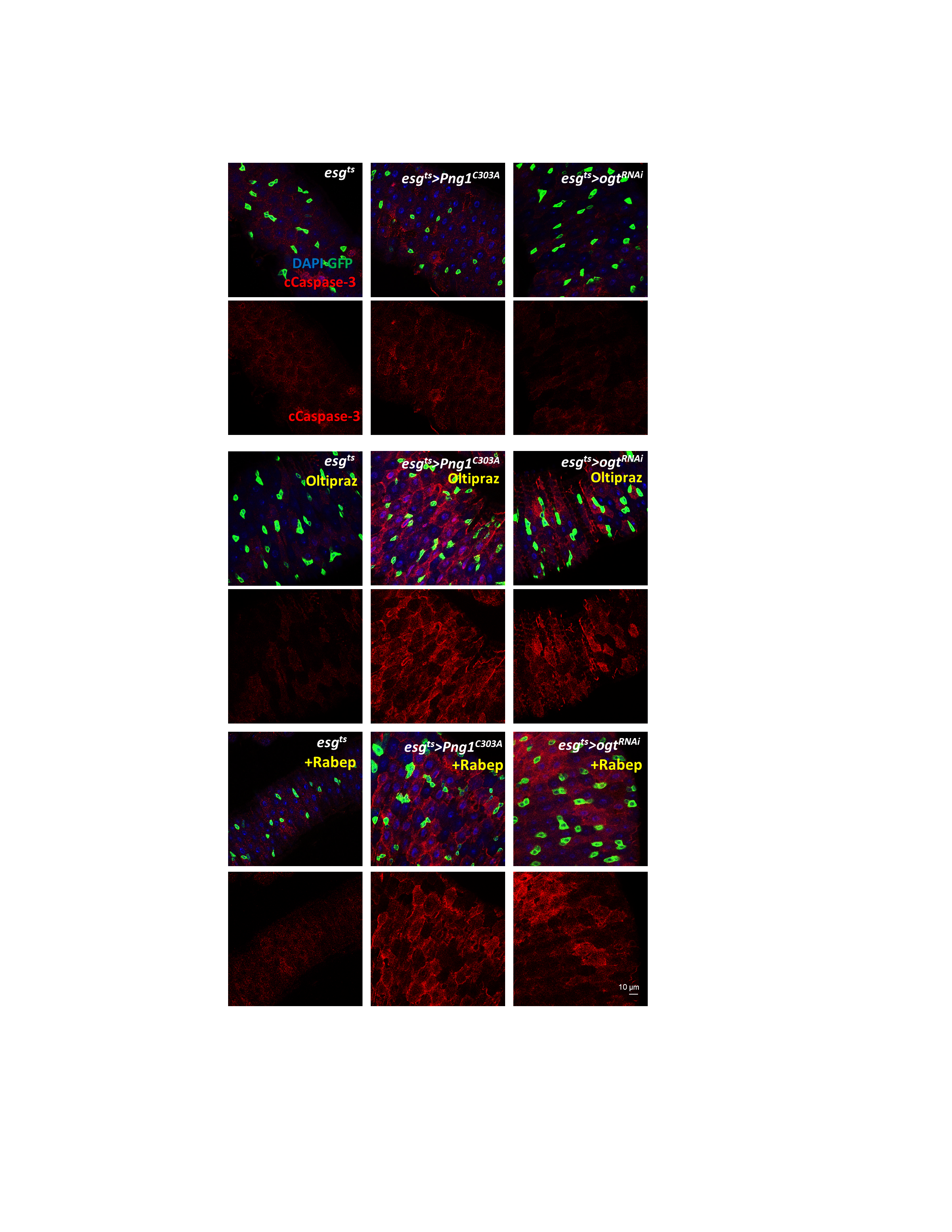

Supplement: S3 Fig — Immunofluorescence staining images of cCaspase-3 (red) in esg-GFP-positive cells (green) in midgut of Oltipraz or treatment in esgts, esgts>Png1C303A, and esgts>OgtRNAi flies. (TIF) [file pgen.1010128.s003.tif]

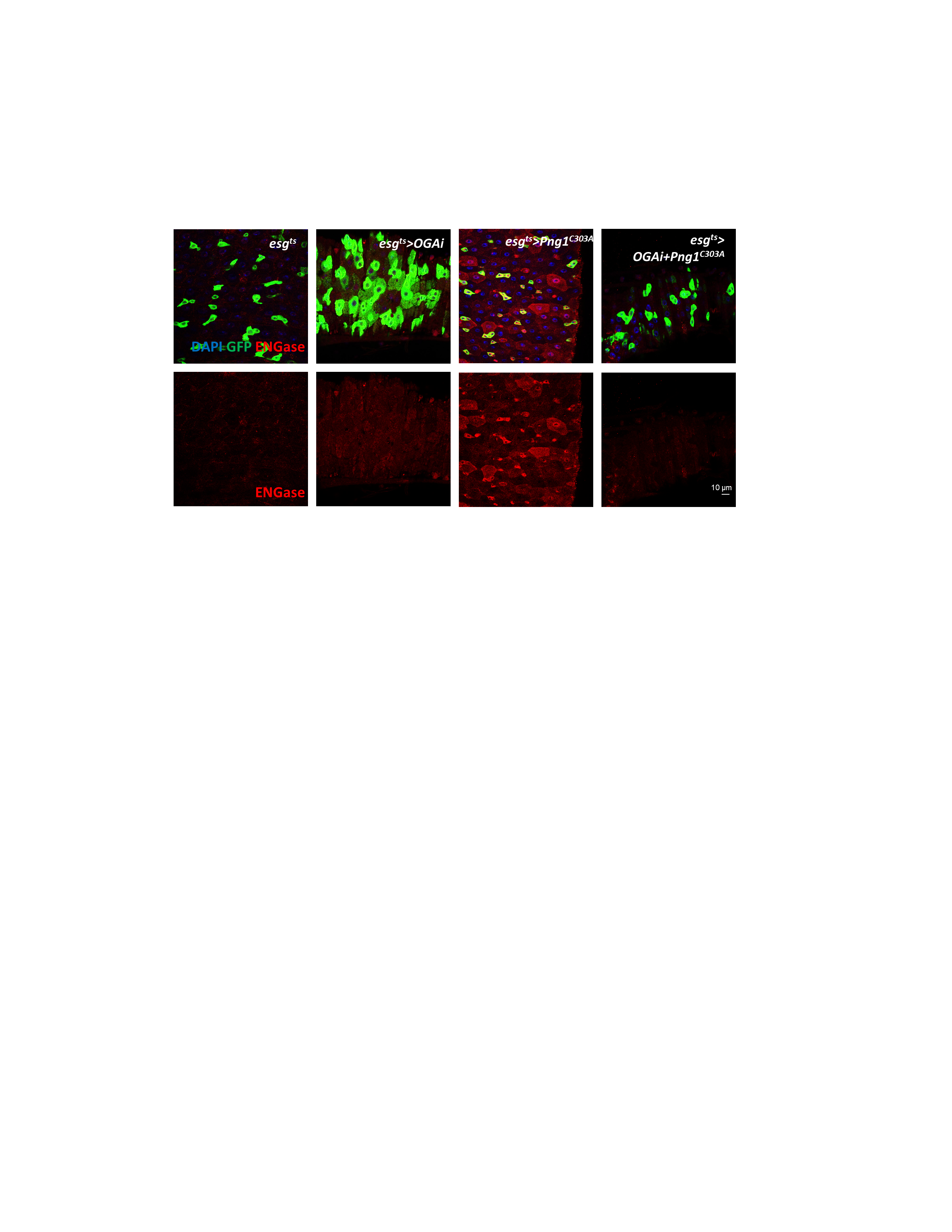

Supplement: S4 Fig — Immunofluorescence staining images of ENGase (red) in esg-GFP-positive cells (green) in midgut of esgts, esgts>OgaRNAi, esgts>Png1C303A and esgts>OgaRNAi+Png1C303A flies. (TIF) [file pgen.1010128.s004.tif]
