# Supplementary material for: Cytosolic O-GlcNAcylation and PNG1 maintain Drosophila gut homeostasis by regulating proliferation and apoptosis
Source: PLoS Genet. 2022 Mar 16;18(3):e1010128. doi: 10.1371/journal.pgen.1010128 (PMC8959174; doi:10.1371/journal.pgen.1010128)
Supplement: S1 Table — (DOCX) [file pgen.1010128.s005.docx]

**Supplemental Information**

**S1 Table. N value of Fig (N = gut, n = cell).**

| **Figure Number** | | **line** | **N** | **n** |
| --- | --- | --- | --- | --- |
| **Fig 1** | **C** | *+/+ ; esg-Gal4,tub-Gal80ts,UAS-GFP/+ ; +/+* (*esg^ts^>+)* | 5 |  |
|  |  | *+/+ ; esg-Gal4,tub-Gal80ts,UAS-GFP/Png1^WT^ ; +/+ (esg^ts^>Png1^WT^)* | 4 |  |
|  |  | *+/+ ; esg-Gal4,tub-Gal80ts,UAS-GFP/Png1^RNAi^ ; +/+ (esg^ts^>Png1^RNAi^)* | 6 |  |
|  |  | *+/+ ; esg-Gal4,tub-Gal80ts,UAS-GFP/+ ; Png1^C303A^/+ (esg^ts^>Png1^C303A^)* | 5 |  |
|  |  | *+/+ ; esg-Gal4,tub-Gal80ts,UAS-GFP/Png1^RNAi^ ; +/+ (esg^ts^>Png1^RNAi^)* | 5 |  |
|  |  | *+/+ ; esg-Gal4,tub-Gal80ts,UAS-GFP/Png1^ex18^ ; +/+ (esg^ts^>Png1^ex18^)* | 5 |  |
|  |  | *+/+ ; esg-Gal4,tub-Gal80ts,UAS-GFP/sxc^7^ ; +/+ (esg^ts^>sxc^7^)* | 5 |  |
|  |  | *+/+ ; esg-Gal4,tub-Gal80ts,UAS-GFP/Ogt^RNAi^ ; +/+ (esg^ts^>Ogt^RNAi^)* | 5 |  |
|  |  | *+/+ ; esg-Gal4,tub-Gal80ts,UAS-GFP/Oga^RNAi^ ; +/+*(*esg^ts^>Ogaa^RNAi^)* | 6 |  |
|  | **D** | *+/+ ; esg-Gal4,tub-Gal80ts,UAS-GFP/+ ; +/+* (*esg^ts^>+)* | 25 |  |
|  |  | *+/+ ; esg-Gal4,tub-Gal80ts,UAS-GFP/Png1^WT^ ; +/+ (esg^ts^>Png1^WT^)* | 12 |  |
|  |  | *+/+ ; esg-Gal4,tub-Gal80ts,UAS-GFP/Png1^RNAi^ ; +/+ (esg^ts^>Png1^RNAi^)* | 29 |  |
|  |  | *+/+ ; esg-Gal4,tub-Gal80ts,UAS-GFP/+ ; Png1^C303A^/+ (esg^ts^>Png1^C303A^)* | 41 |  |
|  |  | *+/+ ; esg-Gal4,tub-Gal80ts,UAS-GFP/Png1^RNAi^ ; +/+ (esg^ts^>Png1^RNAi^)* | 11 |  |
|  |  | *+/+ ; esg-Gal4,tub-Gal80ts,UAS-GFP/Png1^ex18^ ; +/+ (esg^ts^>Png1^ex18^)* | 10 |  |
|  |  | *+/+ ; esg-Gal4,tub-Gal80ts,UAS-GFP/sxc^7^ ; +/+ (esg^ts^>sxc^7^)* | 15 |  |
|  |  | *+/+ ; esg-Gal4,tub-Gal80ts,UAS-GFP/Ogt^RNAi^ ; +/+ (esg^ts^>Ogt^RNAi^)* | 28 |  |
|  |  | *+/+ ; esg-Gal4,tub-Gal80ts,UAS-GFP/Oga^RNAi^ ; +/+*(*esg^ts^>Ogaa^RNAi^)* | 10 |  |
|  | **F** | *+/+ ; +/+ ; Myo1A-Gal4,tub-Gal80ts,UAS-GFP /+ (Myo1A^ts^>+)* | 17 |  |
|  |  | *+/+ ; +/ Png1^WT^; Myo1A-Gal4,tub-Gal80ts,UAS-GFP /+ (Myo1A^ts^>Png1^WT^)* | 10 |  |
|  |  | *+/+ ; +/ Png1^RNAi^ ; Myo1A-Gal4,tub-Gal80ts,UAS-GFP /+ (Myo1A^ts^>Png1^RNAi^)* | 14 |  |
|  |  | *+/+ ; +/ + ; Myo1A-Gal4,tub-Gal80ts,UAS-GFP / Png1^C303A^ (Myo1A^ts^>Png1^C303A^)* | 13 |  |
|  |  | *+/+ ; +/ Png1^RNAi^ ; Myo1A-Gal4,tub-Gal80ts,UAS-GFP /+ (Myo1A^ts^>Png1^RNAi^)* | 16 |  |
|  |  | *+/+ ; +/ Ogt^RNAi^ ; Myo1A-Gal4,tub-Gal80ts,UAS-GFP /+ (Myo1A^ts^>Ogt^RNAi^)* | 24 |  |
|  |  | *+/+ ; +/ Oga^RNAi^ ; Myo1A-Gal4,tub-Gal80ts,UAS-GFP /+ (Myo1A^ts^>Oga^RNAi^)* | 19 |  |
|  | **H** | *+/+ ; esg-Gal4,tub-Gal80ts,UAS-GFP/+ ; +/+* (*esg^ts^>+*) | 4 | 35 |
|  |  | *+/+ ; esg-Gal4,tub-Gal80ts,UAS-GFP/+ ; +/+* (*esg^ts^>+*)+PQ 10 mM | 6 | 42 |
|  |  | *+/+ ; esg-Gal4,tub-Gal80ts,UAS-GFP/Oga^RNAi^ ; +/+*(*esg^ts^>Oga^RNAi^)* | 3 | 34 |
|  |  | *+/+ ; esg-Gal4,tub-Gal80ts,UAS-GFP/+ ; Oga^del.1^/+ (esg^ts^;Oga^del.1^)* | 4 | 34 |
| **Fig 2** | **B** | *+/+ ; esg-Gal4,tub-Gal80ts,UAS-GFP/+ ; +/+* (*esg^ts^>+*) | 5 |  |
|  |  | *+/+ ; esg-Gal4,tub-Gal80ts,UAS-GFP/OGA^RNAi^ ; +/+*(*esg^ts^>OGA^RNAi^)* | 4 |  |
|  |  | *+/+ ; esg-Gal4,tub-Gal80ts,UAS-GFP/+ ; Png1^C303A^/+ (esg^ts^>Png1^C303A^)* | 5 |  |
|  |  | *+/+ ; esg-Gal4,tub-Gal80ts,UAS-GFP/Oga^RNAi^ ; Png1^C303A^/+ (esg^ts^>Oga^RNAi^+Png1^C303A^)* | 5 |  |
|  | **C** | *+/+ ; esg-Gal4,tub-Gal80ts,UAS-GFP/+ ; +/+* (*esg^ts^>+*) | 25 |  |
|  |  | *+/+ ; esg-Gal4,tub-Gal80ts,UAS-GFP/OGA^RNAi^ ; +/+*(*esg^ts^>OGA^RNAi^)* | 17 |  |
|  |  | *+/+ ; esg-Gal4,tub-Gal80ts,UAS-GFP/+ ; Png1^C303A^/+ (esg^ts^>Png1^C303A^)* | 26 |  |
|  |  | *+/+ ; esg-Gal4,tub-Gal80ts,UAS-GFP/Oga^RNAi^ ; Png1^C303A^/+ (esg^ts^>Oga^RNAi^+Png1^C303A^)* | 26 |  |
|  | **E** | *+/+ ; esg-Gal4,tub-Gal80ts,UAS-GFP/+ ; +/+* (*esg^ts^>+*) | 8 | 80 |
|  |  | *+/+ ; esg-Gal4,tub-Gal80ts,UAS-GFP/Oga^RNAi^ ; +/+*(*esg^ts^>Oga^RNAi^)* | 4 | 37 |
|  |  | *+/+ ; esg-Gal4,tub-Gal80ts,UAS-GFP/+ ; Png1^C303A^/+ (esg^ts^>Png1^C303A^)* | 4 | 42 |
|  |  | *+/+ ; esg-Gal4,tub-Gal80ts,UAS-GFP/Oga^RNAi^ ; Png1^C303A^/+ (esg^ts^>Oga^RNAi^+Png1^C303A^)* | 5 | 51 |
|  | **G** | *+/+ ; esg-Gal4,tub-Gal80ts,UAS-GFP/+ ; +/+* (*esg^ts^>+*) | 3 | 47 |
|  |  | *+/+ ; esg-Gal4,tub-Gal80ts,UAS-GFP/Oga^RNAi^ ; +/+*(*esg^ts^>Oga^RNAi^)* | 4 | 43 |
|  |  | *+/+ ; esg-Gal4,tub-Gal80ts,UAS-GFP/+ ; Png1^C303A^/+ (esg^ts^>Png1^C303A^)* | 3 | 33 |
|  |  | *+/+ ; esg-Gal4,tub-Gal80ts,UAS-GFP/Oga^RNAi^ ; Png1^C303A^/+ (esg^ts^>Oga^RNAi^+Png1^C303A^)* | 5 | 71 |
|  | **I** | *+/+ ; esg-Gal4,tub-Gal80ts,UAS-GFP/+ ; +/+* (*esg^ts^>+*) | **5** | **58** |
|  |  | *+/+ ; esg-Gal4,tub-Gal80ts,UAS-GFP/Oga^RNAi^ ; +/+*(*esg^ts^>Oga^RNAi^)* | **4** | **33** |
|  |  | *+/+ ; esg-Gal4,tub-Gal80ts,UAS-GFP/+ ; Png1^C303A^/+ (esg^ts^>Png1^C303A^)* | **5** | **45** |
|  |  | *+/+ ; esg-Gal4,tub-Gal80ts,UAS-GFP/Oga^RNAi^ ; Png1^C303A^/+ (esg^ts^>Oga^RNAi^+Png1^C303A^)* | **6** | **45** |
| **Fig 3** | **B** | *+/+ ; esg-Gal4,tub-Gal80ts,UAS-GFP/+ ; +/+* (*esg^ts^>+*) | 5 | 49 |
|  |  | *+/+ ; esg-Gal4,tub-Gal80ts,UAS-GFP/+ ; OGT/+ (esg^ts^>Myc-OGT)* | 4 | 51 |
|  |  | *+/+ ; esg-Gal4,tub-Gal80ts,UAS-GFP/Png1^RNAi^ ; +/+ (esg^ts^>Png1^RNAi^)* | 3 | 24 |
|  |  | *+/+ ; esg-Gal4,tub-Gal80ts,UAS-GFP/ Png1^RNAi^ ; OGT/+ (esg^ts^>Myc-OGT+ Png1^RNAi^)* | 6 | 51 |
|  | **D** | *+/+ ; esg-Gal4,tub-Gal80ts,UAS-GFP/+ ; +/+* (*esg^ts^>+*) | **6** | **67** |
|  |  | *+/+ ; esg-Gal4,tub-Gal80ts,UAS-GFP/Ogt^RNAi^ ; +/+ (esg^ts^>Ogt^RNAi^)* | **6** | **58** |
| **Fig 4** | **B** | *+/+ ; +/+ ; Myo1A-Gal4,tub-Gal80ts,UAS-GFP /+ (Myo1A^ts^>+)* | **5** |  |
|  |  | *+/+ ; +/ + ; Myo1A-Gal4,tub-Gal80ts,UAS-GFP / Png1^C303A^ (Myo1A^ts^>Png1^C303A^)* | **7** |  |
|  |  | *+/+ ; +/ Oga^RNAi^ ; Myo1A-Gal4,tub-Gal80ts,UAS-GFP /+ (Myo1A^ts^>Oga^RNAi^)* | **5** |  |
|  |  | *+/+ +/ Oga^RNAi^; Myo1A-Gal4,tub-Gal80ts,UAS-GFP / Png1^C303A^ (Myo1A^ts^>Oga^RNAi^ + Png1^C303A^)* | **4** |  |
|  | **C** | *+/+ ; +/+ ; Myo1A-Gal4,tub-Gal80ts,UAS-GFP /+ (Myo1A^ts^>+)* | **24** |  |
|  |  | *+/+ ; +/ + ; Myo1A-Gal4,tub-Gal80ts,UAS-GFP / Png1^C303A^ (Myo1A^ts^>Png1^C303A^)* | 12 |  |
|  |  | *+/+ ; +/ Oga^RNAi^ ; Myo1A-Gal4,tub-Gal80ts,UAS-GFP /+ (Myo1A^ts^>Oga^RNAi^)* | 13 |  |
|  |  | *+/+ +/ Oga^RNAi^; Myo1A-Gal4,tub-Gal80ts,UAS-GFP / Png1^C303A^ (Myo1A^ts^>Oga^RNAi^ + Png1^C303A^)* | **11** |  |
|  | **F** | *+/+ ; +/+ ; Myo1A-Gal4,tub-Gal80ts,UAS-GFP /+ (Myo1A^ts^>+)* | 4 | 55 |
|  |  | *+/+ ; +/ Oga^RNAi^ ; Myo1A-Gal4,tub-Gal80ts,UAS-GFP /+ (Myo1A^ts^>Oga^RNAi^)* | 4 | 54 |
|  |  | *+/+ ; +/ + ; Myo1A-Gal4,tub-Gal80ts,UAS-GFP / Png1^C303A^ (Myo1A^ts^>Png1^C303A^)* | 5 | 51 |
|  |  | *+/+ +/ Oga^RNAi^; Myo1A-Gal4,tub-Gal80ts,UAS-GFP / Png1^C303A^ (Myo1A^ts^>Oga^RNAi^ + Png1^C303A^)* | 4 | 59 |
|  | **H** | *+/+ ; +/+ ; Myo1A-Gal4,tub-Gal80ts,UAS-GFP /+ (Myo1A^ts^>+)* | 4 | **41** |
|  |  | *+/+ ; +/ Oga^RNAi^ ; Myo1A-Gal4,tub-Gal80ts,UAS-GFP /+ (Myo1A^ts^>Oga^RNAi^)* | 5 | **53** |
|  |  | *+/+ ; +/ + ; Myo1A-Gal4,tub-Gal80ts,UAS-GFP / Png1^C303A^ (Myo1A^ts^>Png1^C303A^)* | 4 | **39** |
|  |  | *+/+ +/ Oga^RNAi^; Myo1A-Gal4,tub-Gal80ts,UAS-GFP / Png1^C303A^ (Myo1A^ts^>Oga^RNAi^+Png1^C303A^)* | 4 | **47** |
| **Fig 5** | **B** | *+/+ ; esg-Gal4,tub-Gal80ts,UAS-GFP/+ ; +/+* (*esg^ts^>+*) | 5 |  |
|  |  | *+/+ ; esg-Gal4,tub-Gal80ts,UAS-GFP/+ ; +/+* (*esg^ts^>+*)+*Oltipraz* | 5 |  |
|  |  | *+/+ ; esg-Gal4,tub-Gal80ts,UAS-GFP/Png1^RNAi^ ; +/+ (esg^ts^>Png1^RNAi^)* | 4 |  |
|  |  | *+/+ ; esg-Gal4,tub-Gal80ts,UAS-GFP/Png1^RNAi^ ; +/+ (esg^ts^>Png1^RNAi^)+ Oltipraz* | 5 |  |
|  |  | *+/+ ; esg-Gal4,tub-Gal80ts,UAS-GFP/+ ; Png1^C303A^/+ (esg^ts^>Png1^C303A^)* | 4 |  |
|  |  | *+/+ ; esg-Gal4,tub-Gal80ts,UAS-GFP/+ ; Png1^C303A^/+ (esg^ts^>Png1^C303A^)+Oltipraz* | 5 |  |
|  |  | *+/+ ; esg-Gal4,tub-Gal80ts,UAS-GFP/Ogt^RNAi^ ; +/+ (esg^ts^>Ogt^RNAi^)* | 4 |  |
|  |  | *+/+ ; esg-Gal4,tub-Gal80ts,UAS-GFP/Ogt^RNAi^ ; +/+ (esg^ts^>Ogt^RNAi^)+Oltipraz* | 5 |  |
|  | **C** | *+/+ ; esg-Gal4,tub-Gal80ts,UAS-GFP/+ ; +/+* (*esg^ts^>+*) | 26 |  |
|  |  | *+/+ ; esg-Gal4,tub-Gal80ts,UAS-GFP/+ ; +/+* (*esg^ts^>+*)+*Oltipraz* | 12 |  |
|  |  | *+/+ ; esg-Gal4,tub-Gal80ts,UAS-GFP/Png1^RNAi^ ; +/+ (esg^ts^>Png1^RNAi^)* | 35 |  |
|  |  | *+/+ ; esg-Gal4,tub-Gal80ts,UAS-GFP/Png1^RNAi^ ; +/+ (esg^ts^>Png1^RNAi^)+ Oltipraz* | 26 |  |
|  |  | *+/+ ; esg-Gal4,tub-Gal80ts,UAS-GFP/+ ; Png1^C303A^/+ (esg^ts^>Png1^C303A^)* | 10 |  |
|  |  | *+/+ ; esg-Gal4,tub-Gal80ts,UAS-GFP/+ ; Png1^C303A^/+ (esg^ts^>Png1^C303A^)+Oltipraz* | 6 |  |
|  |  | *+/+ ; esg-Gal4,tub-Gal80ts,UAS-GFP/Ogt^RNAi^ ; +/+ (esg^ts^>Ogt^RNAi^)* | **20** |  |
|  |  | *+/+ ; esg-Gal4,tub-Gal80ts,UAS-GFP/Ogt^RNAi^ ; +/+ (esg^ts^>Ogt^RNAi^)+Oltipraz* | **29** |  |
|  | **E** | *+/+ ; esg-Gal4,tub-Gal80ts,UAS-GFP/+ ; +/+* (*esg^ts^>+*) | **4** | **53** |
|  |  | *+/+ ; esg-Gal4,tub-Gal80ts,UAS-GFP/Png1^RNAi^ ; +/+ (esg^ts^>Png1^RNAi^)* | **5** | **52** |
|  |  | *+/+ ; esg-Gal4,tub-Gal80ts,UAS-GFP/Png1^RNAi^ ; +/+ (esg^ts^>Png1^RNAi^) + Oltipraz* | **7** | **83** |
|  |  | *+/+ ; esg-Gal4,tub-Gal80ts,UAS-GFP/+ ; CncC/+ (esg^ts^>Cncc)* | **3** | **27** |
|  |  | *+/+ ; esg-Gal4,tub-Gal80ts,UAS-GFP/Png1^RNAi^ ; CncC/+ (esg^ts^>Cncc+ Png1^RNAi^)* | **7** | **56** |
|  | **G** | *+/+ ; esg-Gal4,tub-Gal80ts,UAS-GFP/+ ; +/+* (*esg^ts^>+*) | **3** | **32** |
|  |  | *+/+ ; esg-Gal4,tub-Gal80ts,UAS-GFP/Png1^RNAi^ ; +/+ (esg^ts^>Png1^RNAi^)* | **4** | **50** |
|  |  | *+/+ ; esg-Gal4,tub-Gal80ts,UAS-GFP/Png1^RNAi^ ; +/+ (esg^ts^>Png1^RNAi^) + Oltipraz* | **3** | **33** |
|  |  | *+/+ ; esg-Gal4,tub-Gal80ts,UAS-GFP/+ ; CncC/+ (esg^ts^>Cncc)* | **5** | **51** |
|  |  | *+/+ ; esg-Gal4,tub-Gal80ts,UAS-GFP/Png1^RNAi^ ; CncC/+ (esg^ts^>Cncc+ Png1^RNAi^)* | **8** | **73** |
| **Fig 6** | **B** | *+/+ ; esg-Gal4,tub-Gal80ts,UAS-GFP/+ ; +/+* (*esg^ts^>+*) | **4** | **38** |
|  |  | *+/+ ; esg-Gal4,tub-Gal80ts,UAS-GFP/Png1^RNAi^ ; +/+ (esg^ts^>Png1^RNAi^)* | **7** | **72** |
|  |  | *+/+ ; esg-Gal4,tub-Gal80ts,UAS-GFP/Png1^RNAi^ ; +/+ (esg^ts^>Png1^RNAi^)+Rabeprazole* | **7** | **79** |
|  |  | *+/+ ; esg-Gal4,tub-Gal80ts,UAS-GFP/Ogt^RNAi^ ; +/+ (esg^ts^>Ogt^RNAi^)* | **6** | **67** |
|  |  | *+/+ ; esg-Gal4,tub-Gal80ts,UAS-GFP/Ogt^RNAi^ ; +/+ (esg^ts^>Ogt^RNAi^)+Rabeprazole* | **6** | **74** |
|  | **D** | *+/+ ; esg-Gal4,tub-Gal80ts,UAS-GFP/+ ; +/+* (*esg^ts^>+*) | 4 |  |
|  |  | *+/+ ; esg-Gal4,tub-Gal80ts,UAS-GFP/+ ; +/+* (*esg^ts^>+*)+*Rabeprazole* | 3 |  |
|  |  | *+/+ ; esg-Gal4,tub-Gal80ts,UAS-GFP/Png1^RNAi^ ; +/+ (esg^ts^>Png1^RNAi^)* | 5 |  |
|  |  | *+/+ ; esg-Gal4,tub-Gal80ts,UAS-GFP/Png1^RNAi^ ; +/+ (esg^ts^>Png1^RNAi^)+Rabeprazole* | 5 |  |
|  |  | *+/+ ; esg-Gal4,tub-Gal80ts,UAS-GFP/Ogt^RNAi^ ; +/+ (esg^ts^>Ogt^RNAi^)* | 4 |  |
|  |  | *+/+ ; esg-Gal4,tub-Gal80ts,UAS-GFP/Ogt^RNAi^ ; +/+ (esg^ts^>Ogt^RNAi^)+Rabeprazol* | 5 |  |
|  | **E** | *+/+ ; esg-Gal4,tub-Gal80ts,UAS-GFP/+ ; +/+* (*esg^ts^>+*) | 14 |  |
|  |  | *+/+ ; esg-Gal4,tub-Gal80ts,UAS-GFP/+ ; +/+* (*esg^ts^>+*)+*Rabeprazole* | 21 |  |
|  |  | *+/+ ; esg-Gal4,tub-Gal80ts,UAS-GFP/Png1^RNAi^ ; +/+ (esg^ts^>Png1^RNAi^)* | 38 |  |
|  |  | *+/+ ; esg-Gal4,tub-Gal80ts,UAS-GFP/Png1^RNAi^ ; +/+ (esg^ts^>Png1^RNAi^)+Rabeprazole* | 25 |  |
|  |  | *+/+ ; esg-Gal4,tub-Gal80ts,UAS-GFP/Ogt^RNAi^ ; +/+ (esg^ts^>Ogt^RNAi^)* | **16** |  |
|  |  | *+/+ ; esg-Gal4,tub-Gal80ts,UAS-GFP/Ogt^RNAi^ ; +/+ (esg^ts^>Ogt^RNAi^)+Rabeprazol* | **15** |  |
|  | **G** | *+/+ ; esg-Gal4,tub-Gal80ts,UAS-GFP/+ ; +/+* (*esg^ts^>+*) | **21** |  |
|  |  | *+/+ ; esg-Gal4,tub-Gal80ts,UAS-GFP/+ ; Png1^C303A^/+ (esg^ts^>Png1^C303A^)* | **37** |  |
|  |  | *+/+ ; esg-Gal4,tub-Gal80ts,UAS-GFP/ENGase ^RNAi^ ; +/+ (esg^ts^>ENGase^RNAi^)* | **10** |  |
|  |  | *+/+ ; esg-Gal4,tub-Gal80ts,UAS-GFP/ENGase ^RNAi^ ; Png1^C303A^/+ (esg^ts^>Png1^C303A^+ ENGase^RNAi^)* | **30** |  |
|  | **I** | *+/+ ; esg-Gal4,tub-Gal80ts,UAS-GFP/+ ; +/+* (*esg^ts^>+*) | **4** | **39** |
|  |  | *+/+ ; esg-Gal4,tub-Gal80ts,UAS-GFP/+ ; Png1^C303A^/+ (esg^ts^>Png1^C303A^)* | **3** | **38** |
|  |  | *+/+ ; esg-Gal4,tub-Gal80ts,UAS-GFP/ENGase ^RNAi^ ; +/+ (esg^ts^>ENGase^RNAi^)* | **4** | **55** |
|  |  | *+/+ ; esg-Gal4,tub-Gal80ts,UAS-GFP/ENGase ^RNAi^ ; Png1^C303A^/+ (esg^ts^>Png1^C303A^+ ENGase^RNAi^)* | **5** | **52** |
|  | **K** | *+/+ ; esg-Gal4,tub-Gal80ts,UAS-GFP/+ ; +/+* (*esg^ts^>+*) | **5** | **50** |
|  |  | *+/+ ; esg-Gal4,tub-Gal80ts,UAS-GFP/ENGase ^RNAi^ ; +/+ (esg^ts^>ENGase^RNAi^)* | **4** | **55** |
|  |  | *+/+ ; esg-Gal4,tub-Gal80ts,UAS-GFP/+ ; Png1^C303A^/+ (esg^ts^>Png1^C303A^)* | **4** | **46** |
|  |  | *+/+ ; esg-Gal4,tub-Gal80ts,UAS-GFP/ENGase ^RNAi^ ; Png1^C303A^/+ (esg^ts^>Png1^C303A^+ ENGase^RNAi^)* | **5** | **58** |
|  | **M** | *+/+ ; esg-Gal4,tub-Gal80ts,UAS-GFP/+ ; +/+* (*esg^ts^>+*) | **3** | **38** |
|  |  | *+/+ ; esg-Gal4,tub-Gal80ts,UAS-GFP/ENGase ^RNAi^ ; +/+ (esg^ts^>ENGase^RNAi^)* | **3** | **43** |
|  |  | *+/+ ; esg-Gal4,tub-Gal80ts,UAS-GFP/+ ; Png1^C303A^/+ (esg^ts^>Png1^C303A^)* | **3** | **45** |
|  |  | *+/+ ; esg-Gal4,tub-Gal80ts,UAS-GFP/ENGase ^RNAi^ ; Png1^C303A^/+ (esg^ts^>Png1^C303A^+ ENGase^RNAi^)* | **5** | **62** |
| **Fig 7** | **B** | *+/+ ; +/+ ; Myo1A-Gal4,tub-Gal80ts,UAS-GFP /+ (Myo1A^ts^>+)* | **24** |  |
|  |  | *+/+ ; +/+ ; Myo1A-Gal4,tub-Gal80ts,UAS-GFP /+ (Myo1A^ts^>+)+Rabeprazole* | **8** |  |
|  |  | *+/+ ; +/ Png1^RNAi^ ; Myo1A-Gal4,tub-Gal80ts,UAS-GFP /+ (Myo1A^ts^>Png1^RNAi^)* | **16** |  |
|  |  | *+/+ ; +/ Png1^RNAi^ ; Myo1A-Gal4,tub-Gal80ts,UAS-GFP /+ (Myo1A^ts^>Png1^RNAi^)+Rabeprazole* | **13** |  |
|  |  | *+/+ ; +/ Ogt^RNAi^ ; Myo1A-Gal4,tub-Gal80ts,UAS-GFP /+ (Myo1A^ts^>Ogt^RNAi^)* | **23** |  |
|  |  | *+/+ ; +/ Ogt^RNAi^ ; Myo1A-Gal4,tub-Gal80ts,UAS-GFP /+ (Myo1A^ts^>Ogt^RNAi^)+ Rabeprazole* | **9** |  |
|  | **F** | *+/+ ; +/+ ; Myo1A-Gal4,tub-Gal80ts,UAS-GFP /+ (Myo1A^ts^>+)* | **24** |  |
|  |  | *+/+ ; +/+ ; Myo1A-Gal4,tub-Gal80ts,UAS-GFP /+ (Myo1A^ts^>+)+ Oltipraz* | **6** |  |
|  |  | *+/+ ; +/ Png1^RNAi^ ; Myo1A-Gal4,tub-Gal80ts,UAS-GFP /+ (Myo1A^ts^>Png1^RNAi^)* | **18** |  |
|  |  | *+/+ ; +/ Png1^RNAi^ ; Myo1A-Gal4,tub-Gal80ts,UAS-GFP /+ (Myo1A^ts^>Png1^RNAi^)+ Oltipraz* | **17** |  |
|  |  | *+/+ ; +/ Ogt^RNAi^ ; Myo1A-Gal4,tub-Gal80ts,UAS-GFP /+ (Myo1A^ts^>Ogt^RNAi^)* | **35** |  |
|  |  | *+/+ ; +/ Ogt^RNAi^ ; Myo1A-Gal4,tub-Gal80ts,UAS-GFP /+ (Myo1A^ts^>Ogt^RNAi^)+ Oltipraz* | **15** |  |
| **S1 Fig** | **B** | *+/+ ; +/+ ; Myo1A-Gal4,tub-Gal80ts,UAS-GFP /+ (Myo1A^ts^>+)* | **5** |  |
|  |  | *+/+ ; +/ Ogt^RNAi^ ; Myo1A-Gal4,tub-Gal80ts,UAS-GFP /+ (Myo1A^ts^>Ogt^RNAi^)* | **7** |  |
|  |  | *+/+ ; +/ Png1^RNAi^ ; Myo1A-Gal4,tub-Gal80ts,UAS-GFP /+ (Myo1A^ts^>Png1^RNAi^)* | **7** |  |
|  | **C** | *+/+ ; +/+ ; Myo1A-Gal4,tub-Gal80ts,UAS-GFP /+ (Myo1A^ts^>+)* | **5** |  |
|  |  | *+/+ ; +/ Ogt^RNAi^ ; Myo1A-Gal4,tub-Gal80ts,UAS-GFP /+ (Myo1A^ts^>Ogt^RNAi^)* | **7** |  |
|  |  | *+/+ ; +/ Png1^RNAi^ ; Myo1A-Gal4,tub-Gal80ts,UAS-GFP /+ (Myo1A^ts^>Png1^RNAi^)* | **7** |  |
| **S2 Fig** | **B** | *hs-flp, tub-Gal4,UAS-GFP; +; FRT82B, tub-Gal80 / FRT82B* (*FRT82B*) | **4** |  |
|  |  | *hs-flp, tub-Gal4,UAS-GFP;png1^ex18^ +; FRT82B, tub-Gal80 / FRT82B :* (*png1^ex18^*; *FRT82B*) | **6** |  |
|  |  | *hs-flp, tub-Gal4,UAS-GFP;sxc^7^ +; FRT82B, tub-Gal80 / FRT82B :* (*sxc^7^ ; FRT82B*) | **7** |  |
|  | **C** | *hs-flp, tub-Gal4,UAS-GFP; +; FRT82B, tub-Gal80 / FRT82B* (*FRT82B*) | **20*** |  |
|  |  | *hs-flp, tub-Gal4,UAS-GFP;png1^ex18^ +; FRT82B, tub-Gal80 / FRT82B :* (*png1^ex18^*; *FRT82B*) | **36*** |  |
|  |  | *hs-flp, tub-Gal4,UAS-GFP;sxc^7^ +; FRT82B, tub-Gal80 / FRT82B :* (*sxc^7^ ; FRT82B*) | **11*** |  |

* indicated clone number.
